# Interventions to reduce the effects of air pollution and of extreme heat on maternal, newborn, and child health outcomes: a mapping of the literature

**DOI:** 10.7189/jogh.15.04035

**Published:** 2025-02-14

**Authors:** Joe Strong, Rachael Barrett, Ziyaad Surtee, Maggie O’Hare, Francesca Conway, Anayda Portela

**Affiliations:** 1 Department of International Development, London School of Economics and Political Science, Houghton Street, London, UK; 2 World Health Organization Department of Maternal, Newborn, Child and Adolescent Health and Ageing, Geneva, Switzerland

## Abstract

**Background:**

There is an increasing awareness of the ongoing and projected impacts of air pollution and of extreme heat on maternal, newborn, and child health (MNCH) outcomes, showing significant short and long-term health problems. There is a dearth of information available for policy makers on interventions that have been implemented to reduce the impact on MNCH, impeding the integration of action into health planning. This paper presents an inventory of interventions aimed at reducing the effects of these two climate hazards on MNCH.

**Methods:**

We conducted a scoping review of articles published in three databases and grey literature to identify and map interventions implemented to address the impact of air pollution and/or extreme heat on MNCH. Items were included if published between January 2016 and November 2022, regardless of language, and as this is an inventory, regardless of if the intervention was evaluated. Over 32 700 journal items were reviewed for inclusion and a sample of grey literature from web-based searches.

**Results:**

A final inventory of 76 items were included. Interventions identified were primarily based in the Global North (n = 51), with the largest proportion in the USA (n = 17), while 32 items were based in the Global South. Fifty-seven items focused on air pollution, 18 on extreme heat, and one on both. Interventions were categorised in four adapted socioecological components: (i) individual and household interventions (n = 30), (ii) community and service interventions (n = 18), (iii) structural interventions and urban landscape interventions (n = 15), (iv) policy interventions (n = 16). Most items were focused on child health outcomes (n = 65); 61 items were evaluated.

**Conclusions:**

This scoping review maps interventions implemented and proposes a categorisation of these to initiate reflections and dialogue on what has been done and how to start building an evidence base. The review also highlights gaps in interventions and the knowledge base, with most interventions implemented to address air pollution, in the Global North and most addressing child health need. As country programmes seek to address the impact of climate change on MNCH, additional efforts are needed to better understand what has been done, document lessons learned, agree on common outcome measurements and feasible study designs for evaluation to start building the evidence base.

In 2023, the World Health Organization, the United Nations Population Fund, and the United Nations Children’s Fund launched a call to action for key partners in health to work together to raise awareness and commit to addressing the risks of climate hazards on maternal, newborn, and child health (MNCH) [1] (**Box 1**).

Box 1 We use the word ‘women’ throughout this article. This is used as it is reflected in the reports and articles included. The word women is used for anyone who identifies as a woman regardless of sex assigned at birth. We recognise that there are other gender-diverse individuals who do not identify as women but have critical MNCH needs. The intention is not to exclude their experiences but rather to reflect the current lack of data on their experiences.

Studies and reports increasingly demonstrate how the effects of the climate crisis are not uniform across populations, reflecting social and structural determinants of health that are shaped along axes of discrimination and inequality [2]; these effects will have a significant impact on vulnerable populations, including pregnant women, newborns, and children [3,4]. The Working Group II contribution to the Sixth Assessment Report of the Intergovernmental Panel on Climate Change emphasised that women – particularly pregnant women – and children were among the most vulnerable to the climate crisis [5,6].

Both air pollution and extreme heat affect health. The two hazards are interlinked and exacerbate the effects of each other on the climate crisis [7,8]. Projected emission scenarios suggest surface temperatures may rise between 1.1 and 6.4°C in some parts of the world by 2100 [9], and air pollution is increasingly reaching hazardous levels, particularly in large metropoles in the Global South [10]. In addition to being individually significant drivers of adverse health outcomes, the effects of these climate hazards can intersect with and compound existing inequalities and structural disadvantages, for example women’s gendered labour burdens, including physical activities such as household chores and care work [5,11].

Studies highlight how exposure to ambient air pollution among pregnant women and infants increases associated respiratory conditions and is associated with adverse immune, neurological, and cardiometabolic outcomes [12]. Further, exposure to air pollution during pregnancy can be associated with development of pregnancy complications and of adverse birth outcomes such as low birth weight and preterm birth [13,14]. For children, air pollution can lead to short- and long-term impacts on health outcomes [15], including respiratory diseases and a potential impact on cognitive health [16].

Extreme heat can have a variety of potentially significant effects on pregnancy outcomes [17,18]. Various observational studies have reported an association between heat exposure in the weeks prior to birth and increased risks of preterm or stillbirth [19,20]. Newborns are especially vulnerable to extreme heat, due to impaired thermoregulation as a result of immature temperature control systems, which can cause acute and life-threatening health conditions [4]. Moreover, extreme heat can affect food systems, impacting the nutritional status of pregnant and breastfeeding women and children [21,22].

Existing reviews emphasise the epidemiological associations between the climate crisis and adverse MNCH outcomes, highlighting that most available evidence comes from Global North contexts [23–26]. A systematic review of the impact of the high temperatures on birth outcomes found an association between high temperatures and preterm birth, low birth weight, and stillbirths [27]. A meta-analysis investigating maternal exposure to air pollution found that prenatal exposure to major air pollutants during the entire pregnancy can increase the risk of low birth weight [28]. A scoping review that focused on climate change and emergency care in the African continent highlighted evidence of the heightened risks that particularly children and women faced as a result of the climate crisis [29].

The growing body of evidence illustrates the associations between exposures to climate hazards and adverse MNCH outcomes. The evidence particularly on exposure to high temperature or air pollution highlights the essential need to develop interventions that can reduce the effects of climate-related exposures and of air pollution on MNCH. While it is important to understand the different risks faced for MNCH, it is also important to identify evidence-informed actions that can be taken. However, there is a lack of information available in the literature on those interventions that could safeguard MNCH from these two climate hazards. In order to better understand what has been implemented, we conducted a scoping review to establish an inventory of implemented interventions to reduce the impact of exposure to extreme heat and air pollution on MNCH.

## METHODS

Our review was developed to answer the following research question: what interventions are currently being implemented to address the effects of air pollution and extreme heat on MNCH? To answer this, we conducted a scoping review to map the existing literature, adapting the framework developed by Arksey and O’Malley [30], to create an inventory of interventions that aimed to reduce the effects of extreme heat and/or air pollution on MNCH. A scoping review was chosen as the most appropriate method for the aim to map existing work and identify gaps [31]. A review protocol was published and includes further details of the rationale and methods [32].

We searched both the grey and published literature. We included any document which described an intervention, regardless of whether the intervention had been evaluated or not. The selected timeframe reflects publications after December 2015 (signing of the Paris Agreement at COP21) until the time of the search conducted for this review.

Three relevant databases were searched for published literature: Ovid Medline, EMBASE, and Global Health. Articles were de-duplicated and then screened based on title and abstract against the inclusion/exclusion criteria, using Covidence (**Table 1**). Potentially relevant articles were then reviewed based on the full text (Table S1 **in the** **Online Supplementary Document**).

**Table 1:** Inclusion/exclusion criteria

Inclusion
Published between January 2016 and December 2022
Specific to pregnant and postpartum women (six weeks after birth), newborn (birth to 28 d of age) or children (one month up to and including aged 10), parents with newborns or children up to age 10, or a disaggregated sub-sample in a broader intervention for the general population
Focused on interventions implemented to reduce the effects of extreme heat and/or air pollution with or without an evaluation
Any published or grey literature document reporting on the topic of interest including peer-reviewed literature or conference abstracts, editorials, or commentaries published in academic journals or reports available on web sites
Any geographic location
Published in any language
**Exclusion**
No clearly disaggregated sub-sample of pregnant or postpartum women, newborns, and / or children
Interventions aimed to mitigate air pollution where the specified pollutant was not a major health damaging pollutant
Interventions aimed to mitigate air pollution where the specified pollutant was not related to outdoor pollution (limited to indoor air pollution, i.e. cooking stoves)
No clearly described intervention activity; associations between green/blue space and climate hazard exposure were not considered an intervention
Medical treatment protocols
Narrative / literature / scoping / systematic reviews

RB, ZS, MOH, and DM screened articles; FC then re-screened 10% of the excluded articles from the title and abstract screening for quality assurance. JS reviewed the final inventory for a second round of quality checks. Any discrepancies were discussed with all authors to decide whether to include or exclude an article.

The database searches were supplemented by a search for grey literature including of web pages of organisations known to have work related to climate change and other UN agencies and implementing partners with work on MNCH. A call for documents by email was also sent to World Health Organization (WHO) partners; these were screened by RB, ZS, MOH, and DM. The web search was conducted by KM and double checked by AP, FC, and JS.

A template was developed for data extraction and was tested against ten articles (Table S2 in the **Online Supplementary Document**). The development of the template drew on existing work for a rapid scoping review [33]. RB, ZS, MOH, DM, and KM conducted data extraction; JS, AP, and FC reviewed. No quality assessment was conducted as this was a mapping of the literature and could potentially include items that were not evaluated [31].

For the purpose of this scoping review, interventions are treated as one item where they are described and/or evaluated collectively (e.g. an article assessing the combined effects on air pollution of a series of policies over time). For journal articles, this resulted in each article being treated as one item. For grey literature, where case studies were reported separately and were distinct from one another, one report could include multiple items. We followed the PRISMA-ScR guidelines in reporting our findings [34].

## RESULTS

**Figure 1:**
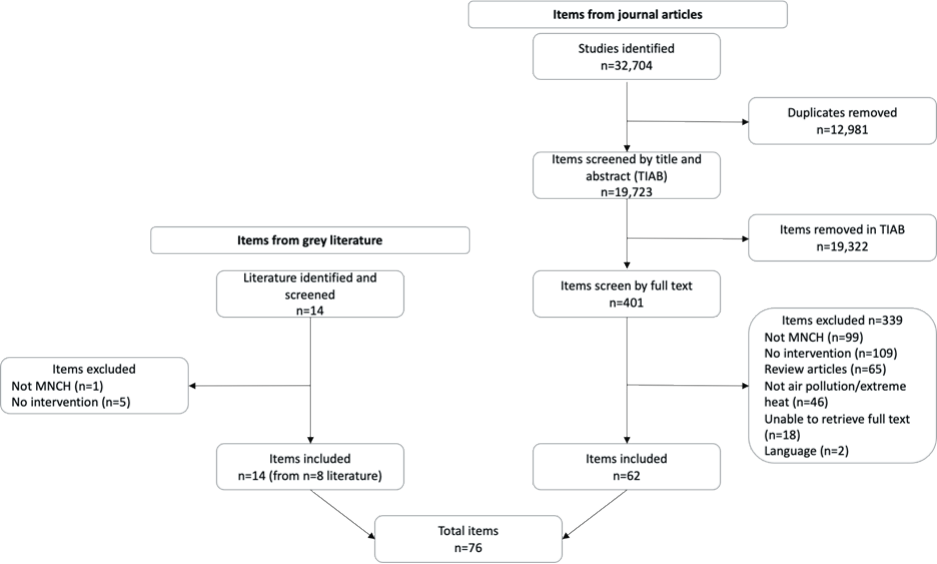
Flow diagram of included items.

**Figure 1** shows the number of items identified in the published and grey literature. A total of 62 interventions were included from journal-based articles, and 14 interventions were included from supplementary grey literature (identified in eight reports). The final inventory includes 76 items from 70 sources, details of each included item are presented in Tables S2–3 in the **Online Supplementary Document**.

**Table 2** summarises key characteristics of the included interventions. Interventions were categorised into four levels.

**Table 2:** Intervention characteristics reported in included items

Intervention description	No. of items included in final inventory
**Climate hazard**	
Air pollution	57
Extreme heat	18
Air pollution and extreme heat	1
Population of interest*	
Children	65
Newborns	12
Pregnant women/postpartum women	10
Parents of newborns/children	10
Levels of intervention*	
Individual and household interventions	30
Community and service interventions	18
Structural interventions	14
Policy interventions	16
**Evaluated interventions**	
Air pollution	55
Extreme heat	5
Air pollution and extreme heat	1

*Multiple areas of interest mean the total ‘n’ is larger than the final inventory of items (n = 76)

The majority of included items focused on air pollution (n = 57, 75%); 18 (23.7%) focused on extreme heat (**Table 2**) and one item focused on both air pollution and extreme heat. After extraction, we categorised the interventions into four levels, using an adapted socio-ecological framework [35,36]:

• individual and household interventions, including interventions aimed directly at the populations of interest or their families

• community and service interventions including interventions designed to impact on community actors, teachers, health workers, etc.

• structural interventions and urban landscape including interventions designed to address buildings like schools or health facilities and urban landscapes

• policy interventions, designed to ensure policies and enabling environments for mitigating climate hazards.

The largest number of included items presented interventions focused on individuals and families (n = 30, 39.5%), followed by communities and service interventions (n = 18, 23.7%). Sixteen items focused on interventions at the policy-level, while fifteen items focused on interventions that addressed school and/or hospital structures or urban landscaping.

Sixty-one (80.3%) of items included an evaluation. No items identified through grey literature were evaluated, although the interventions might have been evaluated in literature not captured in this review. Of evaluations, 55 (90.2%) were for interventions relating to air pollution, five (8.2%) were for interventions relating to extreme heat, and one (1.6%) was for an intervention relating to both air pollution and extreme heat. Sixteen (26.2%) evaluations were randomised controlled trials (RCTs) or other interventions that used randomisation, of which six were evaluations of different outcomes from the same RCT in Mongolia. A further fifteen (24.6%) evaluations used regression analyses, *t* tests, and ANOVA. The remainder included quasi-experimental evaluation designs, modelling of outputs and comparisons pre-post intervention, and mixed methods. One evaluation used qualitative methods.

The majority of items were focused in one country (n = 73, 96.1%) (**Table 3**). The greatest share of these items was based in the Global North (n = 51, 67.1%), with 17 (22.4%) items based in the USA; 32 (42.1%) included items were based in the Global South. Seven items were based in Mongolia, with six of these stemming from the same umbrella intervention but reporting on different outcomes and intervention strands.

**Table 3:** Geographic distribution of interventions identified according to WHO regions*

Region/country	Studies included in final inventory, n (%)
**African Region**	
South Africa	1 (1.3%)
**Eastern Mediterranean Region**	
Iran	3 (3.9%)
Pakistan	1 (1.3%)
United Arab Emirates	1 (1.3%)
**European Region**	
Cyprus	1 (1.3%)
France	3 (3.9%)
Germany	2 (2.6%)
Greece	2 (2.6%)
Italy	2 (2.6%)
Netherlands	1 (1.3%)
Norway	1 (1.3%)
Portugal	3 (3.9%)
Switzerland	2 (2.6%)
Spain	1 (1.3%)
UK	6 (7.9%)
**South-East Asia Region**	
Bangladesh	1 (1.3%)
India	7 (9.2%)
Indonesia	1 (1.3%)
Pakistan	1 (1.3%)
**Region of the Americas**	
Brazil	1 (1.3%)
Canada	4 (5.3%)
Ecuador	1 (1.3%)
Peru	1 (1.3%)
USA	17 (22.4%)
**Western Pacific Region**	
Australia	1 (1.3%)
China	6 (7.9%)
Japan	1 (1.3%)
Mongolia	7 (9.2%)
New Zealand	1 (1.3%)
South Korea	3 (3.9%)
**Global**	1 (1.3%)

WHO – World Health Organization

*Multiple geographies of interest mean the total ‘n’ is larger than the final inventory of items (n = 76).

### Interventions pertaining to individuals and families

The largest proportion of items in the inventory (n = 30, 39.5%) described and/or evaluated interventions that were focused on individuals or families. Twenty-seven of these related to air pollution and three of these related to extreme heat.

#### Air pollution

Interventions with individuals and families used a number of different strategies to encourage behaviours to adapt to the effects of air pollution. The ENRRICH intervention, USA, was a strategic partnership for change with an environmental justice community and included an educational theatrical production and materials for children and families on respiratory health and air pollution [37]. A randomised trial in Cyprus and Greece included warnings for parents on air pollution exposure in homes, to promote adaptation and harm reduction behaviours [38].

In New Zealand, an intervention that measured exposure to air pollution on two walking routes to school, children and carers/parents were encouraged to change their routes to reduce exposure to traffic-related pollution [39]. In Australia, an intervention sought to reduce car idling outside of schools through signage, newsletters, and fact sheets, measuring air pollutant concentrations as the outcome [40]. In an intervention conducted in fifteen primary schools in the UK, air quality monitors were used to measure exposure to air pollution for children and parents participating in travel to school, providing evidence that could be used to inform behaviour change interventions around how to adapt to air pollution exposure [41].

Two interventions focused on the provision of masks to children; one in the UK used children’s ratings of masks as the outcome of interest [42]. The second, in Indonesia, looked at the effectiveness of wearing surgical masks, bandanas, and motorcycle masks using exposure to pollutants as the outcome of interest [43]. An intervention in households in Peru sought to improve the environmental health of people through hygiene education and a child development programme, with indoor air pollution levels measured as the outcome of interest [44]. In Switzerland, a case study with five infants aged 3–14 months used monitors to measure air quality that generated evidence to inform safer sleeping environments [45].

Fourteen interventions focused on measuring the effect of air purifier use on a variety of outcomes. Six interventions all drew from the Ulaanbaatar Gestation and Air Pollution Research Study, Mongolia [46–51]. The randomised controlled trial recruited non-smoking pregnant women, with intervention groups given 1–2 High Particulate Air Filters (HEPA) for their homes to evaluate their impact on MNCH outcomes. Studies measured birth weight, gestational age-adjusted birth weight, birth length, head circumference, gestational age at birth, and size at gestational age [46], air pollution concentration and blood cadmium levels in later pregnancy [47], parent reported scores of behaviours associated with autism [48], parent reported behaviours of children [49], childhood BMI [50], and cognitive outcomes among children [51].

A further eight interventions focused on the use of air purifiers to mitigate the effects of air pollution in the home. All of these eight interventions were particularly focused on fine particulate matter (PM_2.5_) and child health, with interventions also including measurement of coarse particulate matter (PM_10_), ammonia (NH_3_), carbon monoxide (CO), and carbon dioxide (CO_2_). Five interventions were based in households in various contexts in the USA [52–56], two were in Shanghai, China [57,58], and one was based in South Korea [59]. Asthma control and management was the outcome measured for four interventions [52–54,59], and air pollutant concentration was the outcome measured for the other four [55–58].

Pregnant women’s health was the primary area of focus in only four included items. An educational intervention in Iran aimed to increase awareness among pregnant women of the effects of air pollution and reduce barriers to adapting individual behaviour to reduce exposure, including distribution of masks [60]. The intervention measured awareness of air pollution risks as the primary outcome of interest. In South Korea, an eight-part educational programme with pregnant women comprised of different elements of environmental risks, including air pollution. The intervention measured pregnant women’s knowledge and awareness on pollutants, finding that pregnant women in intervention groups had increased knowledge of risks from pollutant exposure than those in the control group [61]. Two separate alert system interventions in Iran used mobile-phone messaging to alert pregnant women to air pollutant exposure and promote adaptation behaviours [62,63]. One of these interventions also included a small-group interview focused on air pollution adaptation behaviours and provision of an educational booklet [62].

#### Extreme heat

Only three interventions pertaining to individuals or families focused on extreme heat. Two grey literature reports focused on the EXTREMA mobile application. The application informs individuals about heat risks, responding to real-time satellite data that geolocates areas of heat risks. It takes into account information on the individual including socio-demographic characteristics, any existing health conditions, and use of medication. An intervention in Paris partnered with the National Observatory of Athens to launch EXTREMA for city residents to stay cool during extreme events [64], and a multi-country launch was initiated across Greece, Spain, Italy, France, and the Netherlands for the app and additional web service dashboard for municipalities to manage information [65]. An intervention with children in China, based in two schools, aimed to raise awareness towards extreme heat and encourage adaptation practices, with the intervention measuring children’s knowledge, awareness, and practice scores [66].

### Interventions pertaining to communities, school and health workers

Eighteen inventory items included interventions that engaged with the community as a whole, including people who work in schools, and people who work in health services. These interventions were primarily focused on air pollution (n = 11), with fewer on extreme heat (n = 7).

#### Air pollution

Community-based interventions included public awareness campaigns and promoting adaptation strategies for air pollution. In Mongolia, an intervention to adapt to the impact of winter air pollution included a large public awareness campaign, policy advocacy, training for health practitioners, and research and evidence generation [67]. The ENRRICH intervention, USA, included school-based adaptation strategies for air pollution (e.g. air filtration), alongside starting a screening clinic in the school for respiratory illnesses associated with exposure. Impact was measured through tracking awareness raising among the populations of interest and respiratory screening programme outcomes [37]. A study in China evaluated the impact of an intervention to raise awareness among child health providers, community stakeholders, and decision makers around the effects of environmental health, using air pollution and other climate health related knowledge measures as the outcome of interest [68].

Many interventions included components aimed at improving awareness among teachers and school-based staff of climate risks. An intervention in Italy alerted teachers of higher air pollution via WhatsApp, after which they were instructed to keep windows closed and use air purifiers to mitigate air pollutant exposure in children’s classrooms [69]. An intervention in Norway installed seventeen low-cost air quality nodes in kindergartens to monitor air quality and children’s exposures to CO, NO, NO_2_, and O_3_, in order for kindergarten staff to plan activities according to exposure risk [70]. In Cyprus and Greece, a randomised trial involved desert dust storm warnings for parents and teachers to reduce pollution exposure among asthmatic children, including guidelines for reducing exposure to PM2_.5_ and PM_10_ in homes and schools alongside installing air cleaners [38].

Further interventions were aimed at changing knowledge and behaviours in schools that could help children adapt to air pollution. An intervention in the USA assessed 36 childcare centres that were randomised into a stepped-wedge protocol of a ‘Greener Cleaning’ educational programme taught by nursing students, community health workers, or asthma coalition members [71]. The intervention had no effect on cleaning behaviours to reduce PM_2.5_ and CO_2_ pollutants. A study in Ottawa, Canada, explored the impact of changing the timings of air ventilation use in schools to account for rush hour traffic, measuring air pollution concentrations and finding a significant reduction for schools that start at 9am, but no reduction for schools that start at 8am [72].

In Portugal, a study in nursery and primary schools evaluated an intervention that focused on various components to improve indoor air quality: awareness, behaviours, activities, technologies, and structures [73]. The intervention saw mitigating effects primarily on CO_2_ levels but no decrease in broader pollutant concentrations to safe levels. Another intervention in Portugal focused on improving school staff knowledge of how to improve indoor air quality through instructions and dedicated guidelines. The intervention included a measure of atopic disease to assess the intervention quality, though the schools saw no reduction in pollutants (PM_2.5_ and PM_10_) [74]. An intervention based in the UK involved training materials co-designed with children, parents of children with asthma, community members, and health professionals on asthma counselling and care that were disseminated among health workers through virtual training sessions and general practitioner surgery engagement settings [75]. This included discussions on asthma and air pollution.

#### Extreme heat

Community-based interventions included education and awareness raising about extreme heat. In France, a national heat-wave plan included educational materials shared among key stakeholders including health workers and the broader populations to help understand the health effects of heat and associated risks [65]. In Maricopa, USA, workshops were conducted to educate the community on heat action plans and urban heat island hotspots, including parks for children [76]. A heat wave forecast intervention in Vietnam aimed to implement early actions to reduce heat-related health impacts such as shelters, cooling centres, and fans with ice tanks [77].

School workers in the USA that participated in the ‘HeatReady’ intervention were trained to identify and adapt to the impacts of school-based extreme heat, measured by knowledge and awareness of heat preparedness [78]. The ‘HeatReady’ intervention aims to build capacity for schools to adapt to the negative impacts of schoolground heat with the aim of improving children’s health and demonstrating that heat safety resources and readiness plans remained under-utilised within schools.

A Heat Action Plan, developed in response to a 2010 heat wave in Ahmedabad, India, included multiple components in its public awareness campaigns and health provider education around the effects of extreme heat and how to adapt to these [76,79]. These also included structural changes to buildings and awareness raising and outreach in schools and with parents and children. A study on the effects of extreme heat evaluated the implementation of heat wave alerts in South Korea, which triggered working hour changes, opening of shade shelters, and deployment of health workers to support climate hazard adaptation [80].

### Interventions based around school and hospital structures and urban landscaping

Fourteen interventions included items focused on the adaptation of school or hospital structures or the urban landscape to reduce the effects of air pollution (n = 4), extreme heat (n = 9), and both (n = 1). All interventions focused on child health as the primary MNCH outcome of interest. One study in the USA analysed the structural renovation of schools, including a new heating, ventilation, and air conditioning system to mitigate both extreme heat and air pollution [76].

#### Air pollution

Included items examined the role of green space-related interventions on children’s health and interventions involving modifications to park and public spaces frequently used by children. Two of these were related to nature-based interventions; planting roadside vegetation in areas frequented by children in the UK [77] and planting green features (trees, wildflower meadows, etc) in elementary school playgrounds in the USA [78]. A further study looked at the effect of the implementation of green and blue spaces in Portugal on children’s health [79].

Items explored the impact of modifying specifically school structures, layouts, and ventilation systems on adapting to the effects of air pollution and extreme heat. In the United Arab Emirates, an intervention that implemented air purifiers, interior renovations with low-emission finishing materials, and new ventilation systems was evaluated based on the presence of air pollution indoors with the aim of improving children’s health [80].

#### Extreme heat

A complex intervention in Paris included mapping and developing ‘cool islands’ – swimming area, water/misting feature, or air-conditioned buildings – to ensure that residents lived within a seven-minute walk of one by 2020. Shading and greenery were developed in schoolyards for cooling under an ‘Urban Oasis’ scheme, and ‘Cool Pathways’ linked the cool islands to minimise discomfort when travelling, including creating tree canopies, solar reflective pavements, or other shading structures [64]. In Cape Town, South Africa, spray parks were installed in recreational spaces in lower income areas to provide cooling services particularly for children [81]. These require less water than swimming pools, which meant they were more feasible for water-stressed cities such as Cape Town.

Three separate interventions in three Canadian cities sought to reduce the effects of extreme heat through infrastructural changes and greening efforts. Interventions included to ‘green’ Anna Street in Québec, an area chosen specifically due to its location near schools to benefit children [82]. The intervention included planting trees, creating rain gardens, and installing permeable surfaces. Shade in areas predominantly used by children was the key intervention focus in Toronto, and in Ontario shade structures, water fountains, and trees were planted to improve shade in parks and playgrounds [82].

Further inventory items included interventions that related to the modification of a broader variety of buildings, including schools and hospitals. In particular, interventions in Ahmedabad [83,84] and Delhi [85], in India, identified in the grey literature focused on ‘cool roofs’ as cost-effective mechanisms to reduce the effects of extreme heat in urban areas. An intervention in Vietnam to mitigate extreme heat included the establishment of cooling centres, as well as household retrofitting and provision of cooling fans with ice tanks for night-time use [81]. Cooling systems such as thermal energy storage tanks and environmentally-friendly refrigerants were also embedded in a high-density city development project in Gujarat, India, including in schools [83].

### Policy-based interventions

Sixteen studies included a policy-based intervention, with seven focused on transportation and traffic-related policies. The majority (n = 15) of these studies were relating to policies to either adapt or mitigate air pollution, with only one related to adaptation to extreme heat.

#### Air pollution

A large proportion (n = 7) of the inventory items assessing policies were focused on transportation and traffic policies. These policies focused on transportation and traffic and were situated in a range of contexts – Brazil [86], Ecuador [87], Germany [88], Japan [89], the UK [90,91], and the USA [92] – the majority of which (n = 5) were based in the Global North. Interventions measured a range of outcomes: respiratory health, premature deaths, adverse birth outcomes, hypertensive disorders, low birth weight, lung function, and air pollution concentrations.

A policy for mandatory motor vehicle inspections (I/M-SP Program) for controlling emissions in São Paulo, Brazil, implemented in 2010 and suspended in 2014, was assessed to see the impact on children’s respiratory health, including asthma [86]. There were no effects of the policy on children’s health. An assessment of vehicular emission controls in Quito, Ecuador, measured the success of the measures through incidences of acute respiratory illness among children, finding the institutionalisation of the policy correlated with a decline in acute respiratory infection between 2000 and 2007 [87]. The Automobile NOx law (1992) that regulated vehicles in Japan through inspections was estimated to reduce air pollution as well as foetal death rates, though no associations were found with the infant mortality rate, low birth weights, or neonatal mortality rate [89]. A further item evaluated a battery of Environmental Protection Agency programmes in the USA, between 1996 and 2008, which aimed to reduce traffic-related air pollution. It measured the impact on newborn health through term birth weight, finding that policies were linked to a decrease in outdoor NO_2_ concentrations and an increase in birth weights [92].

Three studies examined low-emission zones (LEZ) in urban areas (banning air-polluting vehicles from city centres). In Germany, a natural experiment of staggered introduction of LEZs had a small impact on low-birth-weight rates [88]. Two interventions explored the impact of LEZ in London, UK. The LEZ policy was not found to have had an impact when measuring intervention effectiveness through improved lung function among children [90]. However, in a separate study, the LEZ policy was found to reduce the concentrations of harmful air pollutants, particularly NO_2_ [91].

A number of items focused on the adoption of air quality targets and standards to reduce air pollution. An evaluation of global clean air policies focused on the effect on children’s exposure to PM_2.5_ and NO_2_ using premature mortality of children under five as the measure of effectiveness [93]. It estimated the impact of implementing existing regional air quality standards across the globe, finding that implementation of PM_2.5_ limits would significantly reduce premature mortality. Another study assessed current mortality associated with air pollution among infants living in thirteen ‘megacities’ in Bangladesh, China, India, and Pakistan and estimated the impact of mitigation strategies [94]. It found that policies to reduce PM_2.5_ could reduce incidences of acute lower respiratory infection and premature mortality of children in all cities. An analysis of the impact of a supra-municipal plan of measures to reduce air pollution in different sectors such as transport, energy, and industry in Agglomeration of Lausanne-Morges, Switzerland, between 2005 and 2015 that included measures to reduce air pollution estimated that the plan prevented 26 premature deaths among infants and reduced bronchitis and asthma cases [95].

Four items in the USA examined the impact of policies on emissions that went beyond traffic-related pollutants. These included regulating emissions from powerplants [96,97] and electric power stations [98] and the implementation of federal environmental health protections [99]. One study explored the impact of the Clean Air Interstate Rule on newborn and child health, measured by improvements in premature births, infant mortality, gestational length, and birth weight [97]. To evaluate the impact of the US Regional Greenhouse Gas Initiative on newborn and child health, one study measured asthma incidence and term low birth rate, finding the former had the highest change in incidence and the latter the lowest [98]. A further study assessed the impact of the closure of a powerplant in Pennsylvania on newborn health in New Jersey, finding that it was associated with a decrease in low birth weights [96]. A final study provided recommendations based of an intervention entitled Project TENDR, which aimed to examine the links between exposure to toxic chemicals including PM_2.5_ and brain development [99].

#### Extreme heat

One included item evaluated two interventions in India that aimed to assist communities and individuals to adapt to extreme heat: the National Rural Employment Guarantee Act that supported rural workers impacted by temperature fluctuations and the Accredited Social Health Activists that aimed to expand formal health services [100]. These interventions sought to generate behaviour change through policy implementation. The intervention evaluation found that only the health programme was effective in modifying the relationship between extreme temperatures and infant mortality in rural India [100].

## DISCUSSION

Given the increasing awareness of the impact of climate change on MNCH, we conducted this mapping to identify existing literature on interventions that have been implemented to reduce the impact of air pollution and/or extreme heat on MNCH, to contribute to an understanding of the available evidence base and to provide information to countries so that they can start discussing promising interventions and potential solutions [1,3,17,23].

The published literature identified was dominated by the interventions on ambient air pollution (75%); with interventions particularly focused around individuals and families. The few interventions that addressed extreme heat were identified in grey literature, were implemented predominantly in the Global North and related to structural modifications to the urban and environmental landscape and heat health plans. Only one item addressed both extreme heat and air pollution. The causes of air pollution and extreme heat are interrelated, and the multiplicative effects of these two climate hazards on population health, and the potential benefits of combined mitigation efforts, make integrated interventions important [101].

Within MNCH, existing interventions primarily focus on child health as the issue driving the intervention or the outcome of interest. This included interventions aimed at improving air quality in the home as well as air quality relating to school environments and journeys to/from school. Some interventions might have secondary impacts on the health of mothers, pregnant women, parents, carers, or newborns, but these were not made explicit. There was a dearth of interventions that specifically targeted pregnant women and post-partum or lactating women. Interventions designed to mitigate the effects of air pollution or extreme heat on pregnant women typically looked at birth outcomes (gestational length, birth weight, live births).

It is notable that overall fewer interventions were focused in the Global South, given that the effects of extreme heat and ambient air pollution are likely to have an uneven distribution with Global South contexts more significantly impacted by the effects of extreme heat and ambient air pollution [9,10]. Mapping the geographic spread uncovers a hegemony of interventions being conducted in the Global North, particularly the USA. The impacts of climate change are predicted to be more acute in Global South contexts [3,102], and people are made more vulnerable due to less health infrastructure, inequalities in access and health system funding and provision. Thus, it is particularly important to understand contextually effective interventions to mitigate and adapt to the effects of air pollution and extreme heat particularly in the Global South.

The largest proportion of interventions pertained to individuals and families, particularly focusing on household-based changes such as installation of air purifiers. These interventions can have an effect on reducing exposure to air pollution in the home, but are unlikely to change exposures elsewhere. This may also not consider the socio-economic, cultural, and political factors that are important determinants of the health impact. Moreover, scalability may be complicated by the availability and accessibility of technologies that might be particularly unfeasible for people of lower income strata. A similar proportion of interventions were based at the community-level or with school/health workers, were relating to adaptations to school or hospital structures/the urban landscape, or were policy based. For only interventions relating to structural changes extreme heat was the more common focus than air pollution; there was a paucity of policy-based interventions that aimed to reduce the effects of extreme heat. Health systems remained largely overlooked across interventions, despite the critical role that these play in managing the effects of the climate crisis [103].

This mapping – through a scoping review of the literature – highlights the current dearth of research that accounts for potential resource and infrastructural barriers to mitigation and adaptation strategies. These barriers may be unequal across contexts and limit the potential applicability of different strategies in different contexts. This includes engaging with indigenous models of protection and health care, which are shown to improve quality of care delivery for MNCH when provided alongside biomedical care [104]. This includes the linking of approaches to adapting to climate crisis hazards [105].

A scoping review does not assess the quality or effectiveness of interventions [106]. As the body of literature grows, conducting systematic reviews of effectiveness will become important. Various languages were screened in the search (English, French, German, Japanese, Mandarin, Spanish,), however, items identified but published in Polish and Korean were excluded despite being potentially relevant to the review. This was due to the language limitations of the research team. Moreover, English search terms will likely have biased the search towards English-language studies that might lead to a greater focus on English-speaking contexts. Finally, the scoping review was limited by the predominantly biomedical databases used. Future reviews can develop on this body of work by broadening the search strategy to include further databases and grey literature, alongside including more languages. Despite these limitations, the inventory presented in this scoping review is a necessary starting point and future work can build on the gaps identified. Moreover, we recommend that this inventory be updated periodically to reflect increasing policies and interventions in this field. This includes an upcoming World Health Organization content analysis of national heat health plans.

## CONCLUSIONS

This inventory highlights key gaps, as well as an understanding of what interventions have already been done. This is of key critical importance as countries increasingly seek action plans to mitigate and adapt to climate hazards. By mapping interventions, this scoping review is an important step in building the evidence base for future work. The review highlights a number of important considerations for researchers and policymakers. There was a paucity of interventions with health workers, who can have a key role in supporting adaptation efforts towards the effects of climate change, alongside providing care for populations impacted by climate change. There was a notable dearth of journal-based literature on interventions in Global South contexts that are most vulnerable to the effects of the climate crisis. Future research and interventions should focus on the reduction of climate crisis effects in these contexts. Interventions that incorporated or were grounded on contextual and/or indigenous mechanisms for reducing the impact of climate change were sparse and might be especially relevant for ensuring cultural and contextual specificity. More multi-sectoral interventions are needed as well as individual / family-based interventions for heat. Further interventions aimed at strengthening the health system, increasing health worker awareness of climate hazard related care, and overcoming the challenges for these interventions are needed. Much of the literature focused on children, with fewer interventions considering pregnant and postpartum women’s health and well-being. These are critical areas of MNCH and more research would be beneficial. Interventions primarily focused on air pollution concentrations as a key exposure of interest. Further research should interrogate the possible impact of interventions in reducing the effects of air pollution and extreme heat on a broader spectrum of health outcomes within MNCH.

A growing body of work highlights the impacts of climate change on MNCH. Decision makers and programme managers will be interested in implementing interventions to reduce these impacts. This review highlights potential interventions although evaluations are lacking. We highlight the importance of taking stock of what has been done and starting dialogue to discuss the way forward and how to build an evidence base. Discussion can focus on how interventions can be adapted, monitored and evaluated, with lessons learned being documented and shared.

## Additional material


Online Supplementary Document
