# Supplementary material for: Interventions to reduce the effects of air pollution and of extreme heat on maternal, newborn, and child health outcomes: a mapping of the literature
Source: J Glob Health. 2025 Feb 14;15:04035. doi: 10.7189/jogh.15.04035 (PMC11826960; doi:10.7189/jogh.15.04035)
Supplement: Online Supplementary Document [file jogh-15-04035-s001.pdf]

Online Supplementary Files

Supplementary File 1

Search Terms used for database Ovid Medline, EMBASE, and Global Health

Search terms for MNCH:

Construct	Search Terms
Maternal	Maternal OR antenatal OR ante-natal OR prenatal OR pre-natal OR gestati* OR childbirth OR birth OR intrapartum OR obstetric* OR labo?r OR pregnancy OR postpartum OR post-partum OR puerper* OR perinatal OR maternity OR birth attendant OR skilled birth OR f?tal.
Newborn	Newborn OR new-born OR new born OR postnatal OR post-natal OR neonatal OR low birthweight OR low birth-weight OR pre?term OR prematur* OR post?term OR breastfeeding OR breast-feeding OR lactat* OR formula-feeding.
Child	Child* OR childhood OR infant OR postnatal OR under five OR under-five OR underfive OR under 5 OR p?ediatric* OR growth OR infant feeding OR infant nutrition.
Combination of groups	Vulnerable population*

Search terms for climate hazards:

Construct	Search Terms
High ambient temperatures	Heat OR Extreme heat OR High Temperature* OR Environmental Temperature* OR Heat Exposur* OR Heat stress* OR Ambient Temperature* OR Occupational

	Heat Exposure* OR Heatwave* OR Summer OR Humid* OR global warming OR global heating OR greenhouse effect OR greenhouse gas
Air pollution	Air quality OR Air pollution OR Air degradation OR particulate matter OR air pollutant* OR PM10 OR dust OR wildfire OR forest fire OR pollutant* OR aerosol*

Search terms for interventions:

Construct	Search Terms
Interventions or actions	Intervention* OR respons* OR adaptation* OR adaptation strateg* Or measur* OR action OR program* OR programm* OR communit* OR behaviour change* OR home-based OR school-based OR community-based OR Provision OR service deliver* OR service organiz* OR essential services OR maintain* OR mitigation strateg* OR helpline* OR digital OR telemedicine OR tele-health OR policy OR cooling center* OR cooling centre* OR health warning* OR early warning* OR architect* OR landscape* OR solution*

Data extraction codebook column headers

- Author(s)
- Year of publication
- Journal or other type of publication
- Time of data collection (year), or source of data
- Country(ies)and site within country(ies)

- Objective of study
- Study design and analysis method
- Targeted population(s) (maternal and/or newborn and/or child)
- List of the actions taken to mitigate the impacts of high ambient temperatures or ambient air pollution on MNCH.
- Any other relevant extraction topics.

Supplementary File 2: Summary table of included peer-reviewed studies reporting on interventions on climate hazards (n=63)

Author, year [country]	Area of climate change [specifics]	Aim/objective(s)	Population of interest	Focus of intervention [specifics]	Intervention description	Method of evaluation / data analysis	Outcomes measured / of interest	Summary of findings on MNCH / intervention outcome
Implementing the US air quality standard for PM2.5 worldwide can prevent millions of premature deaths per year.<br><br>(Giannadaki, Lelieveld and Pozzer 2016)<br><br>Global	Air pollution [PM2.5]	To evaluate the implementation of air quality standards for PM2.5 globally	Children [1-10]	Adoption of local or national air quality targets and standards	Air quality standards implemented for PM2.5, including: EU directives on clean air, the US Environmental Protection Agency standards, the Canadian Environmental Protection Act, the Clean Air Initiative Asia (primary focus on China), and air quality standards in Colombia, Chile, Ecuador, El Salvador, Mexico, Puerto Rico, Dominican Republic, Argentina, and Bolivia, and further countries with no specific clean air policies.	Epidemiological modelling	Premature mortality of children under five	To evaluate the intervention, the authors used a high-resolution global atmospheric chemistry model combined with epidemiological concentration response functions to investigate premature mortality attributable to PM2.5 in adults ≥30 years and children <5 years.<br><br>Assuming full implementation of PM2.5 reduction policies, it is estimated global premature mortality is reduced by 9%, with reductions in China of 16%, Pakistan of 34%, Bangladesh of 41%, and the US by 4%. Application of EU limits on PM2.5 to China could reduce premature mortality by 31% and 13% in India.
Premature mortality attributable to PM2.5 exposure and future policy roadmap for ‘airpocalypse’ affected Asian megacities<br><br>(Maji, Arora and Dikshit 2018)	Air pollution [PM2.5]	To assess the mortality associated with air pollution and provide an estimation of the benefits of various air pollution mitigation strategies across 13 megacities	Children [1-10]	Adoption of local or national air quality targets and standards	An estimation of the impact of the following policies: (a) the goals of Air Pollution Prevention and Control Action Plan target (APPCAP) for China; (b) current policies (CP) and best practice emission control (BPEC) scenarios and (c) Interim Targets (ITs) and Air Quality Guidelines (AQG) for $PM_{2.5}$ for all megacities.	Regression analysis	Acute lower respiratory infection, premature mortality	In China, acute lower respiratory infection (ALRI) for infants contributed to 0.27 thousand premature deaths due to air pollution. In India, this was 1.3 thousand premature deaths. In Bangladesh, it was 201 premature deaths. In Pakistan, it was 4.85% of total mortality.<br><br>For China, Air Pollution Prevention and Control Action Plan will be able to reduce premature deaths significantly. Furthermore, $PM_{2.5}$ concentrations will reduce in CP and BPEC scenarios in 2030 but premature mortality would increase. In CP, $PM_{2.5}$-related deaths will increase by

Author, year [country]	Area of climate change [specifics]	Aim/objective(s)	Population of interest	Focus of intervention [specifics]	Intervention description	Method of evaluation / data analysis	Outcomes measured / of interest	Summary of findings on MNCH / intervention outcome
China, India, Bangladesh, Pakistan								14–30%, whereas in BPEC scenarios the deaths will increase by 3–14% in the Chinese megacities in 2030. In India, the $PM_{2.5}$-related deaths, in CP scenario, would increase on average by 39.32% in 2025 and by 100% in 2040. Under BPEC mitigation scenario, the deaths will increase by 20.5% and 47.75% in five megacities. The highest increase of $PM_{2.5}$-related deaths in CP scenario has been observed in Dhaka (85%) and Karachi (75%) compared to the base year.
Localized real-time information on outdoor air quality at kindergartens in Oslo, Norway using low-cost sensor nodes.<br><br>(Castell, Schneider et al. 2018)<br><br>Norway	Air pollution [CO, NO, NO2, O3]	To evaluate the provision of effective air quality information to kindergartens	Children [1-10]	Alert systems and/or monitoring for high air pollution for parents, carers, schools and adoption of preventative behaviours	Installed 17 low-cost air quality nodes in kindergartens, plus a data fusion technique that generated a map of air quality in the general area from the nodes' merged data, providing real time air quality information and allowing kindergarten staff to plan activities accordingly	Monitoring air quality	Attitudes towards monitoring air quality	Parents and other stakeholders were engaged in focus groups, with general support for increased information on air quality in kindergartens. Children's health due to exposure to air quality not evaluated.
Mitigating the health effects of desert dust storms through exposure reduction approaches: The LIFE MEDEA asthma study	Air pollution [PM2.5, PM10]	The MEDEA Asthma study aims to assess the impact of behavioural interventions during desert dust storms on the morbidity of asthmatic schoolchildren	Children [1-10]	Alert systems and/or monitoring for high air pollution for parents, carers, schools and adoption of preventative behaviours	Children were randomised in three parallel groups: (a) No Intervention (controls) (b) Outdoor Intervention (c) Outdoor and Indoor Intervention.<br><br>The intervention includes timely DDS warnings to parents' and teachers'	Randomized trial	Air pollution exposure	No evaluation or results

Author, year [country]	Area of climate change [specifics]	Aim/objective(s)	Population of interest	Focus of intervention [specifics]	Intervention description	Method of evaluation / data analysis	Outcomes measured / of interest	Summary of findings on MNCH / intervention outcome
(Kouis, Michailidi et al. 2019)<br><br>Cyprus, Greece					smartphones and communication of animated exposure reduction guidelines while in the houses and classrooms of third group participants, particle air-cleaners have also been installed.			
Effectiveness of a Protocol to Reduce Children's Exposure to Particulate Matter and NO2 in Schools during Alert Days.<br><br>(Zauli-Sajani, Marchesi et al. 2022)<br><br>Italy	Air pollution [PM1, PM2.5, PM10, CO2, NO2]	To test the effectiveness of applying a protocol in the event of alert days	Children [1-10]	Alert systems and/or monitoring for high air pollution for parents, carers, schools and adoption of preventative behaviours	An alert system was used to forecast high PM10 days (above EU limit value) and teachers alerted the day before using Whatsapp to enact a protocol the following (alert) day, including opening classroom door (to prevent CO2 build up), keeping windows closed (except during morning break), air purifier use.	Monitoring air quality	Air pollutant concentrations	Indoor and outdoor mean pollutant concentrations measured on alert and non-alert days. The system correctly forecast 80% of exceedances of the PM10 limit value but generated three false alarms. Lower Indoor/Outdoor ratios of all pollutants were observed on alert days compared to non-alert days, PM10 had the highest absolute difference. Positive feedback from teachers and students regarding ease of application.
A behavioral strategy to minimize air pollution exposure in pregnant women: a randomized controlled trial.<br><br>(Araban, Tavafian et al. 2017)<br><br>Iran	Air pollution	The aim of this study was to evaluate the effectiveness of a theory-based educational program to change pollution exposure behaviour in pregnant women.	104 pregnant women	Alert systems for air pollution episodes and adoption of preventative behaviours	Pregnant women (18-35, no previous pregnancy complications, owning a mobile phone) in the intervention group were given a one hour small-group motivational interview focused on air pollution prevention behaviors, an educational booklet on air pollution (air quality in Tehran, dangers to fetus posed by AP, motivating and	Randomized controlled trial	Alert systems for air pollution episodes and adoption of subsequent preventative behaviours	At the time of follow-up assessment (2 months post intervention), the intervention was found to increase air pollution preventative behaviors among pregnant women in the cohort.

Author, year [country]	Area of climate change [specifics]	Aim/objective(s)	Population of interest	Focus of intervention [specifics]	Intervention description	Method of evaluation / data analysis	Outcomes measured / of interest	Summary of findings on MNCH / intervention outcome
					encouraging statements to prompt behavior change), and a daily small message service for one month regarding daily air quality and encourage preventative behavior (SMS)			
Effectiveness of a theory-based mobile phone text message intervention for improving protective behaviors of pregnant women against air pollution: a randomized controlled trial.<br><br>(Jasemzadeh, Khafaie et al. 2018)<br><br>Iran	Air pollution	To investigate a mobile phone text message intervention for improving protective behaviours against air pollution among pregnant women	Pregnant women	Alert systems for air pollution episodes and adoption of preventative behaviours	Randomized controlled trial of an extended parallel process model-based mobile phone text message intervention. 2 to 7 short messages were sent to participants; the more high levels of air pollution, the more messages were sent. Content were developed by health ministries.	Randomized controlled trial	Pollution protection behaviours	Evaluation looked at air pollution protective behaviours between groups.<br><br>There was statistically significant difference between perceived severity, response efficacy, self-efficacy, and behaviour in the two groups. However, there was no statistically significant difference between perceived susceptibility, before and after intervention.<br><br>Results supported the effectiveness of mobile phone text messaging to promote protective behaviours of pregnant women during periods of high air pollution.
Improving knowledge about children's environmental health in Northwest China.<br><br>(Niu, Qu et al. 2015)	Air pollution	To identify policy maker opinions and attitudes towards children's environmental health	Children [1-10]	Awareness raising and education among policymakers	Child health providers, community stakeholders and decision makers were invited to attend, and encouraged to ask others to join. The agenda was focused on outdoor air pollution, with additional sessions providing context for other environmental	Mixed methods	Knowledge among providers and key stakeholders	After the conference, substantially greater self-efficacy was identified for lead, mercury, air pollution and polychlorinated biphenyls (+0.57–0.72 on a 1–5 Likert scale, p = 0.002–0.013), and the scientific knowledge for the role of environment in children's health (+0.58, p = 0.015), and health care provider control (+0.52, p = 0.025) were rated more strongly.

Author, year [country]	Area of climate change [specifics]	Aim/objective(s)	Population of interest	Focus of intervention [specifics]	Intervention description	Method of evaluation / data analysis	Outcomes measured / of interest	Summary of findings on MNCH / intervention outcome
China					exposures to which children are vulnerable.			
When good fences aren't enough: the impact of neighboring air pollution on infant health.<br><br>(DeCicca and Malak 2020)<br><br>United States	Air pollution [PM2.5, SO2, NO2]	To investigate the impact of the Clean Air Interstate Rule on birth outcomes and infant mortality	Newborns Children [1-10]	Emissions reduction policies in other sectors (energy, industry), such as cap-and-trade policies	The Clean Air Interstate Rule, mandated reduction of powerplant emissions in certain US regions, specified emission "budgets" for each state for each type of pollutant, interstate trading program for SO2 and NOx allowances	Quantitative data analysis	Premature birth, infant mortality, gestational length, birth weight	Compared data collected between 1995 and 2013. The found the policy reduced premature birth, particularly among women aged over 35, and a reduction in infant mortality for newborns among pregnant people who experienced "risky pregnancies".<br><br>Female newborns experienced longer gestation, with a 3% reduction in premature births and a 2% and 4% reduction in the proportion of low and very low birth weight, respectively.
Healthy Air, Healthy Brains: Advancing Air Pollution Policy to Protect Children's Health.<br><br>(Payne-Sturges, Marty et al. 2019)<br><br>United States	Air pollution [PM2.5]	To provide policy recommendations for maintaining and strengthening federal environmental health protections	Children [1-10]	Emissions reduction policies in other sectors (energy, industry), such as cap-and-trade policies	Project TENDR (Targeting Environmental Neurodevelopmental Risks), a collaboration of leading scientists, health professionals, and children's and environmental health advocates, points to growing scientific evidence linking exposure to toxic chemicals during early brain development with brain disorders and calls on individuals, industries, and policymakers to reduce these exposures	No evaluation reported	Mitigation of air pollution causes and exposures	Recommendation 1: The US Environmental Protection Agency (EPA) should give greater consideration to the evidence on the effects of air pollutants on neurodevelopment when setting standards for combustion-related air pollutants and when assessing the full cost of the health effects of air pollution.<br><br>Recommendation 2: Strengthen and enforce federal fuel efficiency standards.<br><br>Recommendation 3: Promote and advance clean energy policies that reduce reliance on fossil fuels, including coal, combusted for energy generation and transportation.<br><br>Recommendation 4: Target existing large sources of combustion-related air pollutants for emissions reductions, dramatically reducing exposures in neighbouring communities.<br><br>Recommendation 5: Regional air pollution control agencies across the United States should restrict permitting new sources of

Author, year [country]	Area of climate change [specifics]	Aim/objective(s)	Population of interest	Focus of intervention [specifics]	Intervention description	Method of evaluation / data analysis	Outcomes measured / of interest	Summary of findings on MNCH / intervention outcome
								combustion-related air pollutants in close proximity to residential areas and other sensitive receptors.<br><br>Recommendation 6: Expand air monitoring near locations where children spend time.<br><br>Recommendation 7: Expand research on effectiveness of strategies to mitigate exposures near large sources of combustion-related air pollution that could guide implementation in neighbourhoods close to such sources.<br><br>Recommendation 8: Increase research on the human health effects of ultrafine particles.
Co-Benefits to Children's Health of the U.S. Regional Greenhouse Gas Initiative.<br><br>(Perera, Cooley et al. 2020)<br><br>United States	Air pollution [NOX, CO_2, SO_2, PM2.5]	To assess the co-benefits of climate change mitigation to children	Children [1-10] Newborns	Emissions reduction policies in other sectors (energy, industry), such as cap-and-trade policies	The Regional Greenhouse Gas Initiative (RGGI) is the United States' first regional market-based regulatory program designed to reduce greenhouse gas emissions from the electric power sector	Health impact assessment	Asthma, term low birth weight	Uses the U.S. Environmental Protection Agency's Benefits Mapping and Analysis Program (BenMAP) tool.<br><br>Asthma had the largest change in incidence, at 537 cases avoided in the study area between the year 2009 and the year 2014.<br><br>TLBW (term low birth weight) had the lowest change in incidence, at a total of 56 incidences avoided.
The impact of environmental regulation on fetal health: evidence from the shutdown of a coal-fired power plant located upwind of New Jersey.	Air pollution [SO_2]	To assess the impact of shutting down a power station on foetal heath	Newborns	Emissions reduction policies in other sectors (energy, industry), such as cap-and-trade policies	Portland Generating Station (located in Pennsylvania) was shut down by the US Environmental Protection Agency after analyses identified it as the sole pollution source impacting air quality downwind in New Jersey. Powerplant shut in June	Regression analysis	Low birth weight, pre-term birth	A difference in differences analysis is used to examine the impact on birth outcomes from the shutdown of the powerplant in New Jersey. Shutting down powerplant reduced likelihood of LBW by 0.89 percentage points (especially within 60miles downwind), and preterm birth by 2.83 percentage points.

Author, year [country]	Area of climate change [specifics]	Aim/objective(s)	Population of interest	Focus of intervention [specifics]	Intervention description	Method of evaluation / data analysis	Outcomes measured / of interest	Summary of findings on MNCH / intervention outcome
(Yang and Chou 2018)<br><br>United States					2014, now only runs as a "peak plant",			
Let's talk air pollution-it's everyones responsibility<br><br>(Parker, Whitehouse et al. 2021)<br><br>United Kingdom	Air pollution	To empower local health professionals with skills and knowledge to talk about and education people on air pollution	Children [1-10]	Health worker patient communication, education and risk counselling	Training materials were created in conjunctional with an environmental charity and the local council, and co-designed with children and young people, parents of children with asthma, community members, and healthcare professionals. These were the disseminated through webinars, virtual training, direct patient context, GP surgery engagement sessions	Knowledge exchange	Discussions on asthma	110 children with asthma have had an air pollution discussion, and over 250 HCPs have been trained up to have these vital discussions.
The Use of a Quasi-Experimental Study on the Mortality Effect of a Heat Wave Warning System in Korea.<br><br>(Heo, Nori-Sarma et al. 2019)<br><br>South Korea	Extreme heat	To examine the use of a heat wave warning system on the mortality effect of heat	Newborns Children [1-10]	Heat (early) warning system of extreme heat events	Heat wave alerts are issued by forecasts for regions where daily maximum temperature is expected to be 33 °C or above for 2 or more consecutive days based on the alert system. One common threshold temperature of 33 °C is used for every region.<br><br>When alert is announced, workplace breaks change, heat-related illness surveillance systems are operated, shade shelters are opened, and health	Regression analysis	All cause and respiratory mortality	They used a quasi-experimental study design with difference in difference models to examine mortality effects.<br><br>The heat wave warning system reduced all cause or respiratory mortality for children aged 0-19, which was particularly caused by changes among children under 5.

Author, year [country]	Area of climate change [specifics]	Aim/objective(s)	Population of interest	Focus of intervention [specifics]	Intervention description	Method of evaluation / data analysis	Outcomes measured / of interest	Summary of findings on MNCH / intervention outcome
					care workers are deployed to help vulnerable sub-populations			
Air pollution exposure in walking school bus routes: A New Zealand case study<br><br>(Dirks, Salmond and Talbot 2018)<br><br>New Zealand	Air pollution	To explore whether reductions in exposure to air pollution from traffic can be achieved in Auckland	Children [1-10]	Managing individual risk through behaviour change outdoors to reduce exposure to air pollutants	Exposure to air pollution on different walking school bus routes were analysed in an urban area of Auckland, New Zealand. Portable P-Trak ultrafine particle monitors and portable carbon dioxide monitors were used to measure the various pollutant levels on either side of the road to determine the effectiveness of walking on the less congested side (during morning rush hour)	Quantitative data analysis	Air pollution levels	The study found that air pollution was higher on more congested sides of the streets, recommending that walking school buses travel on quieter sides of the streets during each rush hour
A factorial cluster-randomised controlled trial combining home-environmental and early child development interventions to improve child health and development: rationale, trial design and baseline findings.	Air pollution [CO]	To present baseline findings of a cluster-randomised controlled trial to evaluate the impact of an integrated home-environmental intervention package	Children [1-10]	Managing individual risk through behaviour change outdoors to reduce exposure to air pollutants	The study implemented a 2 × 2 factorial design trial applying two interventions individually and in combination: i) an environmental health package comprising a certified ICS, kitchen sink and hygiene education (IHIP); and ii) an early child development programme (ECD).	Randomized controlled trial	Personal exposure to pollutants	They measured indoor PM2.5 and CO concentrations stationary from the kitchen environments and personal exposure (CO only) before the installation of the ICS.<br><br>Indoor and personal 24-h CO air pollution measurements in our study meet WHO guidelines, but kitchen $PM_{2.5}$ levels exceed the threshold of 25 μg/m^3 recommended by the WHO (213 μg/m^3).

Author, year [country]	Area of climate change [specifics]	Aim/objective(s)	Population of interest	Focus of intervention [specifics]	Intervention description	Method of evaluation / data analysis	Outcomes measured / of interest	Summary of findings on MNCH / intervention outcome
(Hartinger, Nuño et al. 2020)<br><br>Peru								
Can changing the timing of outdoor air intake reduce indoor concentrations of traffic-related pollutants in schools?<br><br>(MacNeill, Dobbin et al. 2016)<br><br>Canada	Air pollution	To determine the impact of modifying the timing of the heating, ventilation, and air conditioning (HVAC) systems on indoor air pollution	Children [1-10]	Managing individual risk through behaviour change outdoors to reduce exposure to air pollutants	Changing the timings of when HVAC systems were switched on to not correspond to rush hours in schools in Ottawa	Regression analysis	Air pollutant concentrations	There was a significant reduction in concentration of most pollutants in the later starting schools (9am), adjusting for outdoor concentrations. There was no significant reduction in earlier starting schools (8am).
Challenges in evaluating PM concentration levels, commuting exposure, and mask efficacy in reducing PM exposure in growing, urban communities in a developing country.<br><br>(Patel, Shibata et al. 2016)<br><br>Indonesia	Air pollution [PM2.5, PM10]	To assess PM exposure in an Indonesian city with no comprehensive air quality monitor. To examine the efficacy of wearing masks and reducing in-transit PM exposures	Children [1-10]	Managing individual risk through behaviour change outdoors to reduce exposure to air pollutants	The effectiveness of wearing surgical masks, bandanas, and motorcycle masks	Quantitative data analysis	PM exposure	Young children (≤ 5) were the most vulnerable age group, and could not reach the safe dosage of PM exposures even when wearing surgical masks.

Author, year [country]	Area of climate change [specifics]	Aim/objective(s)	Population of interest	Focus of intervention [specifics]	Intervention description	Method of evaluation / data analysis	Outcomes measured / of interest	Summary of findings on MNCH / intervention outcome
Reducing car idling at primary schools: an intervention study of parent behaviour change in Perth, Western Australia. (Rumchev, Lee et al. 2021) Australia	Air pollution [PM10, PM4, PM2.5]	To examine the effectiveness of an anti-idling behaviour intervention targeting parents at primary schools	Children [1-10]	Managing individual risk through behaviour change outdoors to reduce exposure to air pollutants	Intervention used two focus group discussions with parents, a low-intensity 4-week anti-idling intervention was developed, comprising onsite signage, four newsletters, and two fact sheets	Pre-post	Air pollutant concentrations	Anti-idling education can be effective in promoting clean travel behaviours and has potential health benefits for school children.
Assessment of the Wearability of Facemasks against Air Pollution in Primary School-Aged Children in London. (Smart, Horwell et al. 2020) United Kingdom	Air pollution	To assess the perceived wearability of three facemasks among children	Children [1-10]	Managing individual risk through behaviour change outdoors to reduce exposure to air pollutants	Primary-school based pilot study testing the wearability, acceptability, feasibility and perceptions of children when wearing commercially available facemasks (3 brands) during breaktime.	Experiment	Ratings of face masks	Main reported issue was hotness while running/difficulty breathing in face masks; children would wear face mask in future in response to poor air quality. Facemasks could fit children better. Pre-questionnaire identified children already identified air pollution as health hazard.
Indoor Air Quality Prior to and Following School Building Renovation in a	Extreme heat Air pollution [CO2, PM2.5, CO]	To evaluate the indoor environment in public schools in the context of an ongoing urban renovation program to investigate the impact of school building	Children [1-10]	Modifications to school structure, layout, and building materials to enhance	Eleven schools were renovated, including a new heating, ventilation, and air conditioning system	Quantitative data analysis	Air pollutant concentrations Extreme warmth	Levels of PM2.5, CO2, CO, temperature are recorded throughout the academic year in pre- and post-renovation schools and descriptive statistics performed. Prior to renovation average school PM2.5 exceeded WHO guidelines 2% of time, post-renovation all school PM2.5 were

Author, year [country]	Area of climate change [specifics]	Aim/objective(s)	Population of interest	Focus of intervention [specifics]	Intervention description	Method of evaluation / data analysis	Outcomes measured / of interest	Summary of findings on MNCH / intervention outcome
Mid-Atlantic School District.<br><br>(Zaeh, Koehler et al. 2021)<br><br>United States		renovation and replacement on indoor air quality		thermal comfort and mitigate extreme heat indoors				under guideline level. For 7 schools with pre- and post-renovation data, linear mixed models show significant reductions in CO_2, PM2.5 and CO after controlling for season. Following renovation, temperatures more frequently fell within the recommended range, with extreme warmth reduced.
Protecting playgrounds: local-scale reduction of airborne particulate matter concentrations through particulate deposition on roadside 'tredges' (green infrastructure).<br><br>(Maher, Gonet et al. 2022)<br><br>United Kingdom	Air pollution [PM10, PM2.5, PM1]	To examine the effect of roadside vegetation on air pollution	Children [1-10]	Nature based solutions: (Re)-greening outdoor areas, especially those frequented by children, including schools and playgrounds	The planting of roadside vegetation	Quantitative modelling	Air pollutant concentrations	School roadside vegetation structures can significantly reduce playground air pollutant concentrations.<br><br>This includes the use of ivy to block air flows that carry pollutants, as well as wester red cedar tredge that has high leaf magnetic loadings (for PM10).<br><br>Leaf deposition was estimated to remove 26-46% of traffic-sourced PM2.5.<br><br>Effects were visible with 'narrow' (~1m) tredge, which is 10% the recommended width.
Effects of trees, gardens, and nature trails on heat index and child health: design and methods of the Green Schoolyards Project.	Extreme heat	To assess children's physical activity levels and interactions with green features at high and moderate temperatures	Children [1-10]	Nature-based solutions: (Re-)greening outdoor areas, especially those frequented by children such as schools and playgrounds	Three elementary schools in Texas were included for having similar proximity to green space. The "intervention" was green space with added features: trees, wildflower meadow, nature trail. The "low-green park" had low amounts of historical	Observational cohort study	Children's shade seeking behaviours	Heat was measured as the combination for air temperature and relative humidity that captures what the temperature feels like.<br><br>Park areas with trees exhibited the lowest heat values and unshaded areas the highest. During September at the intervention park, there was a 9.8 Fahrenheit difference between the canopied playground and unshaded playground, which was the different

Author, year [country]	Area of climate change [specifics]	Aim/objective(s)	Population of interest	Focus of intervention [specifics]	Intervention description	Method of evaluation / data analysis	Outcomes measured / of interest	Summary of findings on MNCH / intervention outcome
(Lanza, Alcazar et al. 2021)<br><br>United States					green features i.e., trees, the "high-green park" had high amounts of historical green features i.e., trees, wildlife habitat garden, nature trail.			between category of "extreme caution" and "danger" levels of heat exposure.<br><br>Children did not always seek cover / shade, e.g., in the high-green park, but did so in the two other parks. It might mean that proximity of park areas shaped children's behaviours as well as how desirous different areas of the parks were.
A Context-Aware Indoor Air Quality System for Sudden Infant Death Syndrome Prevention.<br><br>(De La Iglesia, De Paz et al. 2018)<br><br>Switzerland	Air pollution [CO, CO2]	To establish safer sleeping environments for newborns	Newborns Children [1-10]	Personal real-time air pollution monitoring using portable or home-based sensors	The intervention developed a system that integrates different air quality sensors for the purpose of monitoring air quality in the room where infants sleep, using a context-aware framework that includes more categories of air quality.	Quantitative data analysis	Abnormal air quality	A case study with five infants aged 3-14 months was conducted and found that the system for monitoring air quality significantly reduces the number of false positives around abnormal air quality compared to threshold-based models
Can portable air quality monitors protect children from air pollution on the school run? An exploratory study.<br><br>(Heydon and Chakraborty 2020)<br><br>United Kingdom	Air pollution	To explore the ways in which portable air quality sensors influence user behaviour	Parents / carers Children [1-10]	Personal real-time air pollution monitoring using portable or home-based sensors	Participants at 15 primary schools were asked to use air quality monitors for 2 weeks on the school run before being interviewed about their experiences, perceptions and behaviours during this time	Quantitative data analysis with personal environment al monitors	Exposure to air pollution	The study has demonstrated that personal environment monitors can play a role in protecting children from air pollution on the school run. They are effective at raising awareness about air pollution, disrupting misconceptions about where it does and does not occur, and encouraging users to change their behaviour in an attempt to mitigate and manage the risks. However, their ability to produce lasting and effective behaviour change is stymied by socio-structural constraints.

Author, year [country]	Area of climate change [specifics]	Aim/objective(s)	Population of interest	Focus of intervention [specifics]	Intervention description	Method of evaluation / data analysis	Outcomes measured / of interest	Summary of findings on MNCH / intervention outcome
Assessment of Indoor Air Quality and Cleaning Behaviors in Urban Child Care Facilities<br><br>(Lee, Dressel et al. 2020)<br><br>United States	Air pollution [PM2.5, CO2]	To assess the impact of an educational programme on air quality in child care centres in Wisconsin	Children [1-10]	Provision of educational information (including preventative behaviours) to parents, families, carers, teachers	18 small and 18 large Milwaukee County, Wisconsin child care centers were recruited and randomized in a stepped-wedge protocol to an early or delayed provision of a Greener Cleaning educational program. An internet-enabled consumer-grade multi-variable IAQ monitor was installed in the centers to establish baseline and post-intervention IAQ.	Stepped wedge crossover trial	Cleaning behaviours	The intervention had no effects on cleaning behaviours
Effectiveness Evaluation of a Primary School-Based Intervention against Heatwaves in China.<br><br>(Li, Sun et al. 2022)<br><br>China	Extreme heat	To evaluate the effectiveness of a primary-school based intervention programme against extreme heat	Children [1-10]	Provision of educational information (including preventative behaviours) to children	Two schools selected – one for intervention and one for control. In the intervention school, a series of health education activities were carried out to raise the primary school students' awareness of and ability to respond to climate change and heatwaves from May to September in 2017	Quantitative data analysis	Knowledge, awareness, attitude, and practice towards heat waves and mitigation	The intervention led to increased knowledge, attitudes, and practice.<br><br>In terms of knowledge, the scores for cognition of heat waves, high temperature warning, and hot weather definition increased the most, by 44.7% (95%CI: 35.8%, 53.6%), 34.2% (95%CI: 24.7%, 43.7%), and 33.4% (95%CI: 27.4%, 39.4%), respectively.<br><br>The scores for the awareness of climate change adaptation measures and heatstroke treatment measures increased 14.9% (95%CI: 8.94%, 20.9%) and 13.8% (95%CI: 8.78%, 18.9%).<br><br>For attitude and practice, the scores for willing to learn relevant knowledge and paying attention to weather forecasts increased by 11.9% (95%CI: 6.79%, 16.9%) and 14.8% (95%CI: 8.48%, 21.1%), respectively.

Author, year [country]	Area of climate change [specifics]	Aim/objective(s)	Population of interest	Focus of intervention [specifics]	Intervention description	Method of evaluation / data analysis	Outcomes measured / of interest	Summary of findings on MNCH / intervention outcome
								There was also a positive effect on parent's knowledge, attitude, and practice, with significant changes in scores across all measures.
Indoor air quality and atopic sensitization in primary schools: A follow-up study<br><br>(Rufo, Madureira et al. 2016)<br><br>Portugal	Air pollution [PM2.5, PM10, CO2, CO]	To investigate how indoor air quality changed after applying recommendations from the SINPHONIE guidelines	Children [1-10]	Provision of educational information (including preventative behaviours) to parents, families, carers, teachers	IAQ changed in primary schools after applying indoor air quality recommendations (SINPHONIE-based recommendations), and to explore how these changes influenced allergic sensitization on children. The school staff received instructions on how to improve IAQ in accordance with the dedicated guidelines. Atopy status was assessed in children attending the participating classrooms by skin prick tests and exhaled nitric oxide. A follow-up sampling campaign was performed in 2014–2015 in the same schools.	Quantitative data analysis	Air pollutant concentrations, atopic disease	SINPHONIE guidelines was particularly successful in reducing PM2.5 and PM10 in primary schools of Porto. Nevertheless, the schools failed to reduce the levels of other IAQ pollutants, as well as the prevalence of atopic disease.
Evaluation of low-cost mitigation measures implemented to improve air quality in nursery and primary schools	Air pollution [CO2, CO, NO2, CO3, CH2O, PM1, PM2.5, PM10]	To evaluate of mitigation measures implemented in nursery and primary schools to improve air quality	Children [1-10]	Provision of educational information (including preventative behaviours) to parents, families, carers, teachers	IAP mitigation: less to the most expensive and complex: Type I—raising awareness; Type II—behavioural changes; Type III—changes in products/materials and places of activities; Type IV—technical and technological changes;	Quantitative data analysis	Air pollutant concentration	In general, pollutant concentrations measured after the implementation of low-cost mitigation measures were significantly lower, mainly for CO_2. However, mitigation measures were not always sufficient to decrease the pollutants' concentrations till values considered safe to protect human health.

Author, year [country]	Area of climate change [specifics]	Aim/objective(s)	Population of interest	Focus of intervention [specifics]	Intervention description	Method of evaluation / data analysis	Outcomes measured / of interest	Summary of findings on MNCH / intervention outcome
(Sá, Branco et al. 2017)<br><br>Portugal				Modifications to school structure and layout; use of building materials with low emissions profiles to reduce exposure during school<br><br>Transportation and traffic policies around schools to reduce exposure to traffic-related air pollutants	Type V—structural changes			
HeatReady schools: a novel approach to enhance adaptive capacity to heat through school community experiences, risks, and perceptions.<br><br>(Shortridge, Walker Vi et al. 2022)<br><br>United States	Extreme heat	To improve understandings of heat perceptions, reactions and actions, and heat safety recommendations of key stakeholders, and to identify themes from expert stakeholder responses to gauge the effectiveness of their heat preparedness	Children [1-10]	Provision of educational information (including preventative behaviours) to schools	HeatReady Schools are those that are increasingly able to identify, prepare for, mitigate, track, and respond to the negative impacts of schoolground heat	Qualitative data analysis	Knowledge and awareness of heat preparedness	Findings demonstrate that 1) heat safety resources are available but not fully utilized within the schools, 2) expert opinions support extreme heat readiness plans accounting for site-specific needs, particularly education, and 3) students are negatively impacted by extreme heat, whether direct or indirect, both inside and outside the classroom.

Author, year [country]	Area of climate change [specifics]	Aim/objective(s)	Population of interest	Focus of intervention [specifics]	Intervention description	Method of evaluation / data analysis	Outcomes measured / of interest	Summary of findings on MNCH / intervention outcome
Strategic Partnerships for Change in an Environmental Justice Community: The ENRRICH Study.<br><br>(Spencer-Hwang, Soret et al. 2016)<br><br>United States	Air pollution	To assess potential adverse health impacts and develop interventions and mitigation plans	Children [1-10] Parents / carers	Provision of educational information (including preventative behaviours) to children<br><br>School-based respiratory health screening in high pollution areas	Community partnership (University, community residents, local CBO, school) organised participatory research in elementary school next to railyard in disadvantaged neighbourhood. Presented an educational theatrical production, provided educational materials for children/families on respiratory health and air pollution, respiratory screening clinic in school, community identification of school-based mitigation strategies (air filtration system, vegetation border)	Multiple methods	Respiratory screening programme, awareness raising	74% of 1,440 children who attended a mobile respiratory clinic were given parental consent to be screened.<br><br>Children who attended school near a railyard had greater airway obstruction than those seven miles away.
Effect of Pro-Environmental Prenatal Education Program on Pregnant Women's Environmental Health Awareness and Behaviors based on the Protection Motivation Theory.<br><br>(Kim and Jeong 2022)	Air pollution	To examine the impact of a pro-environmental prenatal education programme on pregnant women's environmental health awareness	Pregnant women	Provision of educational information to pregnant women (including preventative behaviours)	The programme consisted of eight parts within four sessions on: the environment and pregnancy, environmental toxin, effects of EDCs, airborne pollutants, water pollutant, radio-electronic exposure, and pro-environmental health behaviours during pregnancy	Quasi-experimental	Pregnant women's education on pollutants	The intervention had positive results across all educational areas of the programme compared to pregnant women in the control group

Author, year [country]	Area of climate change [specifics]	Aim/objective(s)	Population of interest	Focus of intervention [specifics]	Intervention description	Method of evaluation / data analysis	Outcomes measured / of interest	Summary of findings on MNCH / intervention outcome
South Korea								
The effect of training based on health belief model (HBM) in preventing exposure to polluted air in pregnant women<br><br>(Pormosayebi, Shamsi et al. 2018)<br><br>Iran	Air pollution	To determine the effect of education using health Belief Model on improving the prevention of exposure to polluted air in pregnant women	Pregnant women	Provision of educational information to pregnant women (including preventative behaviours)	Educational intervention aimed on increasing awareness among pregnant women of the effects of polluted air and reduce barriers to timely action to prevent exposure, including distribution of masks	Quasi-experimental	Awareness of air pollution risks	Awareness of the risks of air pollution in the intervention group increased significantly, as well as the main performance score based on an assessment of perceived barriers.
Heat, infant mortality, and adaptation: evidence from India.<br><br>(Banerjee and Maharaj 2020)<br><br>India	Extreme heat	To examine the impact of extreme heat during pregnancy on infant mortality and check if public interventions can serve as effective adaptation strategies	Newborns Pregnant women	Public employment, health, and social care programs as modifiers of the relationship between heat and infant mortality	Reviews two policy interventions: National Rural Employment Guarantee Act (NREGA) aims to support rural workers impacted by temperature fluctuations; Accredited Social Health Activists (ASHA) aimed to expand access to formal health services	Quantitative data analysis	Infant mortality	Only the health programme is effective in modifying the temperature-infant mortality relationship in rural India.
The zero impact of the Vehicle Inspection Program on public health in Sao Paulo, SP.<br><br>(Araujo and Araujo 2020)	Air pollution [PM2.5, CO, CO2]	To assess the impact of mandatory motor vehicle inspections for controlling emission of pollutants (2010-2014) on health	Children [1-10]	Transportation and traffic policies to reduce ambient concentrations of traffic-related air pollutants	Mandatory motor vehicle inspection for controlling emission of pollutants (I/M-SP program), implemented in 2010 and suspended in 2014	Interrupted time series	Children's respiratory health [respiratory diseases, asthma]	There were no significant effects of the policy on children's respiratory health

Author, year [country]	Area of climate change [specifics]	Aim/objective(s)	Population of interest	Focus of intervention [specifics]	Intervention description	Method of evaluation / data analysis	Outcomes measured / of interest	Summary of findings on MNCH / intervention outcome
Brazil								
Health benefits of a reduction of PM10 and NO2 exposure after implementing a clean air plan in the Agglomeration Lausanne-Morges.<br><br>(Castro, Künzli and Götschi 2017)<br><br>Switzerland	Air pollution [PM10, NO2]	To estimate the health impacts associated with the reduction of small particle matter exposure	Newborns Children [1-10]	Transportation and traffic policies to reduce ambient concentrations of traffic-related air pollutants	A super-municipal policy of a combination of measures to reduce air pollution in transport, energy, and industry.	Health impact assessment	Premature deaths, bronchitis, asthma symptoms	On the impact of the policy between 2005 and 2015, the authors estimate that a reduction of exposure to PM_{10} prevented 26 premature deaths (equivalent to around 290 years of life lost including 30 years of working life lost) among infants below 1 year old and adults age 30 and older. 150 prevalent cases of bronchitis and 1,000 asthma symptom days in children were also prevented.
Air pollution control and the occurrence of acute respiratory illness in school children of Quito, Ecuador.<br><br>(Estrella, Sempértegui et al. 2019)<br><br>Ecuador	Air pollution [PM2.5]	To evaluate the effect of a city-wide five-year air pollution control programme in Quito	730 Children [1-10]	Transportation and traffic policies to reduce ambient concentrations of traffic-related air pollutants	A comparison of 616 children attending school in 2000 and 730 children attending school in 2007 to explore the impact of the city wide policies on air pollution, including: the formation of the Metropolitan Atmospheric Monitoring Network Quito, vehicular emission controls (established in 2002).	Quantitative data analysis	Acute respiratory illness	The rate ratio for the association COHb greater than the safety risk level of 2.5% and acute respiratory illness decreased by 67.5%.<br><br>The average percentage of children with COHb over the safety level of 2.5% decreased by 92% between the study years.<br><br>The annual frequency of ARI per child declined 46% between the 2000 and 2007 cohorts.
The effect of low emission zones on air pollution and infant health.	Air pollution [CO]	To analyse the effectiveness of low-emissions zones in different cities over time on newborn health.	Newborns	Transportation and traffic policies to reduce ambient concentrations	The staggered introduction of low-emission zones in Germany are analysed as a natural experiment	Natural experiment	Low birth weight	Observed improvements to air quality as a result of LEZ are small; stage 1 bans of most pollutant vehicles reduced PM10 particulates by 1.5-2.5%, while more restrictive LEZ led to a 3-4% reduction. These changes are estimated as too small

Author, year [country]	Area of climate change [specifics]	Aim/objective(s)	Population of interest	Focus of intervention [specifics]	Intervention description	Method of evaluation / data analysis	Outcomes measured / of interest	Summary of findings on MNCH / intervention outcome
(Gehrsitz 2017)<br><br>Germany				of traffic-related air pollutants				to impact newborn health, measured as low birth weight.
Impact of the London low emission zone on children's respiratory health: A sequential yearly cross sectional study 2008-2014<br><br>(Griffiths, Mudway et al. 2016)<br><br>United Kingdom	Air pollution [PM2.5, NOx, NO2, PM10]	To examine the relationship between pollutant exposures and lung function following the introduction of the low-emission zone in London	2,297 Children [1-10]	Transportation and traffic policies to reduce ambient concentrations of traffic-related air pollutants	Introduction of Low Emission Zone in London	Analysis of pollutant exposures	Lung function among children	They found no evidence of a change in the lung function of children over this period, despite small improvements in air quality in highly polluted urban areas during the implementation of London's LEZ.
Particulate air pollution, birth outcomes, and infant mortality: Evidence from Japan's automobile emission control law of 1992<br><br>(Inoue, Nunokawa et al. 2020)<br><br>Japan	Air pollution [NOx, SO2, Ox, SPM]	To investigate the impact of the Automobile NOx Law of 1992 on fetal and infant health outcomes	Newborns	Transportation and traffic policies to reduce ambient concentrations of traffic-related air pollutants	The Automobile NOx law (1992) was a largescale automobile regulation through motor vehicle inspections	Quantitative data analysis	Foetal dearth rate, infant mortality rate, low birth weight, neonatal mortality rate	The policies significantly reduced air pollution. The estimated 3.5% reduction in foetal death rate attributable to the regulation fully accounts for the observed 2.4% decrease in foetal death rate. No other newborn and infant (up to 1yo) health outcomes were significantly associated with the regulation.

Author, year [country]	Area of climate change [specifics]	Aim/objective(s)	Population of interest	Focus of intervention [specifics]	Intervention description	Method of evaluation / data analysis	Outcomes measured / of interest	Summary of findings on MNCH / intervention outcome
Impact of London's low emission zone on air quality and children's respiratory health: a sequential annual cross-sectional study<br><br>(Mudway, Dundas et al. 2019)<br><br>United Kingdom	Air pollution [PM2.5, PM10]	To investigate the impact of London's low emission zones on air quality and children's respiratory health	Children [1-10]	Transportation and traffic policies to reduce ambient concentrations of traffic-related air pollutants	Introduction of a low emission zone in London	Quantitative data analysis	Air pollutant concentrations, COPD	Conducted a sequential annual cross-sectional study of 2164 children aged 8-9 attending primary schools in central London.<br><br>The percentage of children living at addresses exceeding the EU limit value for annual NO_2 (40 μg/m^3) fell from 99% (444/450) in 2009 to 34% (150/441) in 2013.<br><br>The study found a reduction in NO_2 at both roadside (median −1·35 μg/m^3 per year; 95% CI −2·09 to −0·61; p=0·0004) and background locations (−0·97; −1·56 to −0·38; p=0·0013), but not for PM_{10}. The effect on $PM_{2·5}$ was equivocal.<br><br>The study found no association between postbronchodilator FEV_1 and annual residential pollutant attributions. FVC was inversely correlated with annual NO_2 (−0·0023 L/μg per m^3; −0·0044 to −0·0002; p=0·033) and PM_{10} (−0·0090 L/μg per m^3; −0·0175 to −0·0005; p=0·038).
Assessing the effectiveness of vehicle emission regulations on improving perinatal health: A population-based accountability study<br><br>(Willis, Hill et al. 2020)	Air pollution [NO2]	To evaluate infant health risks associated with maternal residences near highways during pregnancy	Pregnant women<br>Newborns	Transportation and traffic policies to reduce ambient concentrations of traffic-related air pollutants	Between 1996 and 2008, state and federal governance introduced multiple traffic-related emissions policies, including banning lead in gasoline; new emissions standards for diesel engines, SUVs, heavy duty vehicles, light duty trucks, Energy Policy Act 2005, National Low Emissions Vehicles program (1998-200), Renewable Fuel Standard Program (2007)	Quantitative data analysis	Term birth weight	The impact of traffic related policies in the period 1996-2009 was evaluated using geocoded maternal and child health data (n=394,346 living within 300m of highway [intervention group]) and compared against daily average NO2 concentrations and health data on term birth weight. From 1996-2009, outdoor NO2 decreased by 51.3% and birthweight increased by 0.3g per year (matched models) over the same period

Author, year [country]	Area of climate change [specifics]	Aim/objective(s)	Population of interest	Focus of intervention [specifics]	Intervention description	Method of evaluation / data analysis	Outcomes measured / of interest	Summary of findings on MNCH / intervention outcome
United States								
Green and blue spaces and lung function in the Generation XXI cohort: a life course approach<br><br>(Almeida, Paciência et al. 2022)<br><br>Portugal	Air Pollution [NO2]	To explore the relationship between childhood lung function and "Residential Normalised Difference Vegetation Index" which included all urban vegetation, including both public and private gardens and green spaces.	Children [1-10]	Urban landscape management	Implementation of urban green and blue spaces. The included blue spaces were naturally occurring major seas and rivers (both smaller natural and man-made urban water sources were discounted due to size).	Quantitative data analysis	Children's lung function	Green spaces, or higher NDVI within 100m of residences led child occupants to experience higher lung function (according to lung function measures employed by study). Therefore, urban children can be seen as benefitting. Blue spaces were not found to have the same impact. NO2 was not found to have a mediating effect.
The effect of portable HEPA filter air cleaner use during pregnancy on fetal growth: The UGAAR randomized controlled trial.<br><br>(Barn, Gombojav et al. 2018)<br><br>Mongolia	Air pollution [PM2.5]	To assess the effect of portable high efficiency particulate air (HEPA) filter air cleaner use during pregnancy on foetal growth.	Pregnant women Newborns	Utilisation of air cleaners, filters, purifiers in homes during pregnancy	The Ulaanbaatar Gestation and Air Pollution Research (UGAAR) study is a single-blind randomized controlled trial conducted in Ulaanbaatar, Mongolia. 540 non-smoking pregnant women recruited at ≤18 weeks gestation were randomized to an intervention (1-2 air cleaners in homes from early pregnancy until childbirth) or control (no air cleaners) group.	Randomized controlled trial	Birth weight (primary outcome), gestational age-adjusted birth weight, birth length, head circumference, gestational age at birth, small for gestational age (all secondary)	The intervention found no significant difference in birth weight between the control and intervention groups.<br><br>In a sub-group of term births, the intervention was associated with greater birth weight.
The effect of portable HEPA filter air cleaners on indoor PM2.5 concentrations and second hand tobacco	Air pollution [PM2.5]	To assess the effectiveness of reducing PM2.5 and second hand smoke exposure	Pregnant women	Utilisation of air cleaners, filters, purifiers in homes during pregnancy	The Ulaanbaatar Gestation and Air Pollution Research (UGAAR) study is a single-blind randomized controlled trial conducted in Ulaanbaatar, Mongolia.	Randomized controlled trial	Air pollution concentration, blood cadmium in late pregnancy	PM2.5 concentrations were lower in intervention cersus control groups

Author, year [country]	Area of climate change [specifics]	Aim/objective(s)	Population of interest	Focus of intervention [specifics]	Intervention description	Method of evaluation / data analysis	Outcomes measured / of interest	Summary of findings on MNCH / intervention outcome
smoke exposure among pregnant women in Ulaanbaatar, Mongolia: The UGAAR randomized controlled trial.<br><br>(Barn, Gombojav et al. 2018)<br><br>Mongolia					540 non-smoking pregnant women recruited at ≤18 weeks gestation were randomized to an intervention (1-2 air cleaners in homes from early pregnancy until childbirth) or control (no air cleaners) group.			
Portable HEPA filter air cleaner use during pregnancy and children's autistic behaviors at four years of age: The UGAAR randomized controlled trial<br><br>(Enkhbat, Gombojav et al. 2022)<br><br>Mongolia	Air pollution [PM2.5]	To explore the impact of reducing particulate matter exposure during pregnancy on the development of autistic traits in children at age four.	540 Pregnant women Children [1-10]	Utilisation of air cleaners, filters, purifiers in homes during pregnancy	540 non-smoking women who were <18 weeks gestation were enrolled; half were given one or two HEPA cleaners depending on the size of the home and the control group received no HEPA cleaners. Follow up occurred during pregnancy and included 478 children.	Randomized controlled trial	Parent-reported autism scores in children	They founded limited evidence of any association between reduction of indoor particulate matter during pregnancy and parent-reported autism scores in four-year-old children.
Portable HEPA filter air cleaner use during	Air pollution [PM2.5]	To quantify the impact of reducing particulate matter air pollution during	540 Pregnant women Children [1-10]	Utilisation of air cleaners, filters, purifiers in homes	540 non-smoking women who were <18 weeks gestation were enrolled; half were given	Randomized controlled trial	Parent-reported behaviours of children	There was no association found between reducing indoor air pollution during pregnancy on parent-reported behaviours of children

Author, year [country]	Area of climate change [specifics]	Aim/objective(s)	Population of interest	Focus of intervention [specifics]	Intervention description	Method of evaluation / data analysis	Outcomes measured / of interest	Summary of findings on MNCH / intervention outcome
pregnancy and children's behavior problem scores: a secondary analysis of the UGAAR randomized controlled trial<br><br>(Enkhbat, Gombojav et al. 2021)<br><br>Mongolia		pregnancy on behavioural problem scores among children		during pregnancy	one or two HEPA cleaners depending on the size of the home and the control group received no HEPA cleaners. Follow up occurred during pregnancy and included 478 children.			
Portable HEPA filter air cleaner use during pregnancy and children's body mass index at two years of age: The UGAAR randomized controlled trial<br><br>(Tamana, Gombojav et al. 2021)<br><br>Mongolia	Air pollution [PM2.5]	To evaluate the impact of HEPA filtration in homes throughout pregnancy (but not after) on childhood BMI outcomes	Children [1-10]	Utilisation of air cleaners, filters, purifiers in homes during pregnancy	The Ulaanbaatar Gestation and Air Pollution Research (UGAAR) study is a single-blind randomized controlled trial conducted in Ulaanbaatar, Mongolia. 540 non-smoking pregnant women recruited at ≤18 weeks gestation were randomized to an intervention (1-2 air cleaners in homes from early pregnancy until childbirth) or control (no air cleaners) group.	Randomized controlled trial	Childhood BMI outcomes	Intervention had no significant impact on BMI
Portable HEPA Filter Air Cleaner Use during	Air pollution [PM2.5]	To evaluate the impact of HEPA filtration in homes throughout pregnancy	Children [1-10]	Utilisation of air cleaners, filters, purifiers in homes	The Ulaanbaatar Gestation and Air Pollution Research (UGAAR) study is a	Randomized controlled trial	Cognitive outcomes among children	Cognitive outcomes were assessed in 383 children at 4yrs using a widely accepted measure of cognitive functioning (WPPSI-IV). Use of air cleaners in first trimester

Author, year [country]	Area of climate change [specifics]	Aim/objective(s)	Population of interest	Focus of intervention [specifics]	Intervention description	Method of evaluation / data analysis	Outcomes measured / of interest	Summary of findings on MNCH / intervention outcome
Pregnancy and Children's Cognitive Performance at Four Years of Age: The UGAAR Randomized Controlled Trial.<br><br>(Ulziikhuu, Gombojav et al. 2022)<br><br>Mongolia		(but not after) on childhood cognitive outcomes		during pregnancy	single-blind randomized controlled trial conducted in Ulaanbaatar, Mongolia. 540 non-smoking pregnant women recruited at ≤18 weeks gestation were randomized to an intervention (1-2 air cleaners in homes from early pregnancy until childbirth) or control (no air cleaners) group.			improved cognitive outcomes by 2.5 points, but not statistically significant.
Effects of filtered fresh air ventilation on classroom indoor air and biomarkers in saliva and nasal samples: A randomized crossover intervention study in preschool children<br><br>(Gao, Xu et al. 2019)<br><br>China	Air pollution [PM2.5]	To evaluate the short-term effects of filtered fresh air ventilation on classroom indoor air in preschools	Children [1-10]	Utilising air purifiers and/or adapting ventilation in schools	Fresh air ventilation systems (FAVS) with high efficiency HEPA filter were installed in classroom for 2 continuous school days.	Quantitative data analysis	Air pollutant exposure	Classrooms with air ventilation had significantly lower fine particulate matter than those without. Each 10 μg/m^3 decrease of indoor $PM_{2.5}$ during the 8 school hours in the first intervention day was associated with an average of 1.76% (95% confidence interval (CI) 0.43–3.08%) increase in saliva lysozyme. This percentage increased to 2.41% (95%CI 0.52–4.26%) if related to the $PM_{2.5}$ level in 16 school hours over 2 days of intervention.
School Environmental Intervention to Reduce	Air pollution [PM2.5]	Pilot to examine the impact of a classroom-based air cleaner intervention	Children [1-10]	Utilising air purifiers and/or adapting	18 classrooms were randomized to receive a High Efficiency	Quantitative data analysis	Asthma symptoms	Frequency of asthma symptoms were based on caregiver responses to the questions about daytime and nighttime symptoms in the past 2 weeks, and

Author, year [country]	Area of climate change [specifics]	Aim/objective(s)	Population of interest	Focus of intervention [specifics]	Intervention description	Method of evaluation / data analysis	Outcomes measured / of interest	Summary of findings on MNCH / intervention outcome
Particulate Pollutant Exposures for Children with Asthma<br><br>(Jhun, Gaffin et al. 2017)<br><br>United States		on reductions to air pollutants		ventilation in schools	Particulate Air (HEPA) cleaner			interference with the child's activities in the past 2 weeks based on a validated questionnaire. This pilot study demonstrated that a classroom-based air cleaner intervention significantly reduced classroom levels of two important indoor particulate pollutants; PM2.5 and Black Carbon.<br><br>Among spirometry findings, the air cleaner intervention improved peak expiratory flow by up to 16% (or 0.46 L/s; 95% CI: 0.1, 0.8; p=0.03). There was suggestive evidence of greater decreases in asthma symptoms in the air cleaner group.
Reduction of particulate matter concentrations by local removal in a building courtyard: Case study for the Delhi American Embassy School<br><br>(Vervoort, Blocken and van Hooff 2019)<br><br>India	Air pollution [PM2.5]	To assess the extent to which removal of local PM2.5 can lead to reduced exposure	Children [1-10]	Utilising air purifiers and/or adapting ventilation in schools	Project installed 4 ESP units in varying configurations around a school building/courtyard and measured the impact on PM2.5 levels in courtyard, corridors, staircases.	Quantitative data analysis	Air pollutant concentration	Volume-averaged PM2.5 concentrations in different areas (courtyard, staircase, corridor) and levels of the building. Trialed different configurations of ESP units. ESP units significantly reduced PM2.5 concentrations in corridors by 25.0-34.1%, in courtyard by 25.3-29.7%. Some configurations had significant impact on staircase volumes. PM2.5 reduction decreased on higher floors. No evaluation on health included
Effectiveness of Using Enhanced Filters in Schools and	Air pollution [PM2.5]	To investigate exposure, health, and cost impacts of high efficiency filters in home and schools	Children [1-10]	Utilising air purifiers and/or adapting ventilation in	High efficiency filters were placed in homes and schools	Health impact assessment	Asthma burden, air pollutant concentrations	Replacing inefficient filters with enhanced filters in schools would reduce the $PM_{2.5}$-attributable asthma burden by 13% annually, with higher benefits for more efficient filters. Marginal costs average $63

Author, year [country]	Area of climate change [specifics]	Aim/objective(s)	Population of interest	Focus of intervention [specifics]	Intervention description	Method of evaluation / data analysis	Outcomes measured / of interest	Summary of findings on MNCH / intervention outcome
Homes to Reduce Indoor Exposures to PM2.5 from Outdoor Sources and Subsequent Health Benefits for Children with Asthma<br><br>(Martenies and Batterman 2018)<br><br>United States				schools and family homes				per classroom or $32 per child with asthma per year. In homes, using efficient furnace filters or air cleaners yields 11 to 16% reductions in the asthma burden with an annualized marginal costs of $151–494 per household.
Real-time measurements of PM2.5 and ozone to assess the effectiveness of residential indoor air filtration in Shanghai homes.<br><br>(Barkjohn, Norris et al. 2021)<br><br>China	Air pollution [PM2.5]	To explore the effectiveness of an intervention method to reduce exposure to air pollution in highly polluted urban areas	Children [1-10]	Utilising air purifiers filters and/or adapting ventilation in family homes	Placing air cleaners with HEPA and activated carbon filters in children's bedrooms. Families and children were also instructed to keep bedroom windows and doors closed.	Analysis of air monitor data	Personal exposure to PM2.5	Air cleaners significant reduced indoor concentrations and individual exposure to air pollutants
The impact of household air cleaners on the chemical composition and children's exposure to	Air pollution [PM2.5]	To evaluate the effectiveness of air cleaners on reducing personal exposure to air pollution	Children [1-10]	Utilising air purifiers filters and/or adapting ventilation in family homes	Households provided an air filter (a washable pre-filter to remove large particles, a disposable HEPA filter for smaller particles, and an activated carbon filter	Monitoring air quality and filters	Personal Environmental Monitors (filter mass to determine chemical load)	The functioning air filters reduced levels of PM2.5 in households.

Author, year [country]	Area of climate change [specifics]	Aim/objective(s)	Population of interest	Focus of intervention [specifics]	Intervention description	Method of evaluation / data analysis	Outcomes measured / of interest	Summary of findings on MNCH / intervention outcome
PM2.5 metal sources in suburban Shanghai.<br><br>(Brehmer, Norris et al. 2019)<br><br>China					for retention of some volatile organic compounds) placed in child's bedroom. Functioning and non-functioning air filters were compared.			
Patterns and predictors of air purifier adherence in children with asthma living in low-income, urban households.<br><br>(Kaviany, Brigham et al. 2022)<br><br>United States	Air pollution [PM2.5]	To understand patterns of use and barriers to air purifier interventions among children	Children [1-10]	Utilising air purifiers filters and/or adapting ventilation in family homes	Two portable air purifiers (Rabbit Air™ Minus A1 Model 700 or 780) were provided to participants (paid for by the study). One purifier (Model 780) was placed in the participant's bedroom. The second purifier (Model 700) was placed in another room commonly utilized by the participant (typically the common room/family room). Those in the control group received identical placebo air purifiers with internal air filters removed.	Quantitative data analysis	Asthma control	Use and adherence did not vary significantly by the room the purifiers were in. 70% of participants used the purifier more than 80% of the time. Among participants who used the purifiers most, they did so on the highest settings significantly more than those who used the purifiers least.<br><br>Participants with care takers who had lower income and public insurance used the purifiers less.<br><br>Participants who reported well controlled asthma used the purifiers 16% more than those who perceived their asthma as poorly controlled. There was no significant association between asthma severity and air purifier adherence.<br><br>Use and adherence was associated to time of year, with participants in winter cohort using less, but not associated with reminders. Barriers included purifiers reverting to factory settings.
Effects of indoor air cleaner with filter in	Air Pollution [CO, PM2.5, CO2]	To evaluate the effects of air purifiers on the concentrations of indoor air pollutants and on	Children [1-10]	Utilising air purifiers filters and/or adapting	Thirty asthma school children with asthma were randomly allocated into a control and intervention. Control	Randomised crossover trial	Asthma medication use among children	There was a statistically significant decrease in anti-asthmatic medication taken by children who had an air purifier with a filter. The mean concentration of

Author, year [country]	Area of climate change [specifics]	Aim/objective(s)	Population of interest	Focus of intervention [specifics]	Intervention description	Method of evaluation / data analysis	Outcomes measured / of interest	Summary of findings on MNCH / intervention outcome
children with asthma<br><br>(Lee, Kim et al. 2020)<br><br>South Korea		asthma control in children.		ventilation in family homes	groups were given an air purifier with filter for three weeks, and intervention groups were given an air purifier without a filter. After a 2-week filter change period, the groups were swapped.			PM2.5 was significantly lower in the filter group than non-filter group.
Assessing Impact of Household Intervention on Indoor Air Quality and Health of Children with Asthma in the US-Mexico Border: A Pilot Study.<br><br>(Moreno-Rangel, Baek et al. 2020)<br><br>United States	Air pollution [PM2.5]	To examine the impact of the combined intervention with asthma education and air purifier on indoor air quality and health outcomes	Children [1-10] Parents	Utilising air purifiers filters and/or adapting ventilation in family homes	Community health workers educated children and their parents on asthma control and management. Air purifiers were then installed	Quantitative data analysis	Asthma control and management	The mean $PM_{2.5}$ levels in each place were significantly improved. Overall, they significantly decreased by 1.91 $\mu g/m^3$ on average. All surveys showed better health outcomes; particularly, quality of life for children was significantly improved.
Effectiveness of air purifier on health outcomes and indoor particles in homes of children with allergic diseases in Fresno, California: A pilot study.	Air pollution [PM2.5]	To evaluate the effectiveness of reducing the levels of indoor fine particulate matter using air purifiers on health outcomes in children with asthma and/or allergic rhinitis	Children [1-10]	Utilising air purifiers filters and/or adapting ventilation in family homes	Air purifiers (HEPA filters) installed in living rooms and bedrooms of children with allergic rhinitis in Fresno, California vs a control group.	Control trial	Childhood asthma control	The air purifier intervention reduced indoor $PM_{2.5}$ levels by 43% (p = 0.001).<br><br>Scores in childhood asthma control tests increased for the intervention group but not significantly more for baseline. There was a decrease for the control group.<br><br>Although there was a trend toward a reduction in night time nasal symptom scores in the active group, the difference was not significant during the study period. Individual symptom components, which

Author, year [country]	Area of climate change [specifics]	Aim/objective(s)	Population of interest	Focus of intervention [specifics]	Intervention description	Method of evaluation / data analysis	Outcomes measured / of interest	Summary of findings on MNCH / intervention outcome
(Park, Cheng et al. 2017)<br><br>United States								include symptom scores separated individually for congestion, rhinorrhea, nasal itching, and sneezing, were significantly decreased at week 6 in the active group and these reductions were also observed at week 12 ($p = 0.035$, $p = 0.012$, $p = 0.033$, and $p = 0.025$, respectively).
Effectiveness of portable HEPA air cleaners on reducing indoor PM2.5 and NH3 in an agricultural cohort of children with asthma: A randomized intervention trial.<br><br>(Riederer, Krenz et al. 2021)<br><br>United States	Air pollution [PM2.5]	To examine the effectiveness of HEPA cleaners in the home	Children [1-10]	Utilising air purifiers filters and/or adapting ventilation in family homes	All families received asthma education, and then were randomized into a control and intervention group. Intervention groups received two indoor HEPA air purifiers.	Randomized intervention trial	Air pollutant concentrations	At follow up, HEPA intervention groups had 60% and 42% lower PM2.5 levels in their sleeping and living areas respectively
Effectiveness of portable HEPA air cleaners on reducing indoor endotoxin, PM10, and coarse particulate matter in an agricultural	Air pollution [PM10, PM2.5, NH3]	To examine the effectiveness of HEPA cleaners in the home	Children [1-10]	Utilising air purifiers filters and/or adapting ventilation in family homes	All families received asthma education, and then were randomized into a control and intervention group. Intervention groups received two indoor HEPA air purifiers.	Randomized intervention trial	Air pollutant concentrations	At follow-up, HEPA families had 46% lower (95% CI, 31%-57%) PM_{10} on average than control families.<br><br>HEPA families had 49% (95% CI, 6%-110%) and 89% lower (95% CI, 28%-177%) $PM_{10-2.5}$ at follow-up.

Author, year [country]	Area of climate change [specifics]	Aim/objective(s)	Population of interest	Focus of intervention [specifics]	Intervention description	Method of evaluation / data analysis	Outcomes measured / of interest	Summary of findings on MNCH / intervention outcome
cohort of children with asthma: A randomized intervention trial.<br><br>(Riederer, Krenz et al. 2021)<br><br>United States								
Improving the Indoor Air Quality in Nursery Buildings in United Arab Emirates.<br><br>(Arar and Jung 2021)<br><br>United Arab Emirates	Air pollution	To suggest effective management and improvement measures for indoor air quality in nurseries	Children [1-10]	Modifications to school structure and layout; use of building materials with low emissions profiles to reduce exposure during school	Various structural interventions for nurseries: air purifiers, interior renovation with low-emission finishing materials and new ventilation installation (using natural and mechanical ventilation to increase inflow of outdoor air)	Quantitative data analysis	Indoor air pollution	New ventilation reduced air pollutant concentrations

Almeida, D. Q., I. Paciência, C. Moreira, J. Cavaleiro Rufo, A. Moreira, A. C. Santos, H. Barros and A. I. Ribeiro (2022). "Green and blue spaces and lung function in the Generation XXI cohort: a life-course approach." Eur Respir J **60**(6).
Araban, M., S. S. Tavafian, S. M. Zarandi, A. R. Hidarnia, A. Burri and A. Montazeri (2017). "A behavioral strategy to minimize air pollution exposure in pregnant women: a randomized controlled trial." Environ Health Prev Med **22**(1): 26.
Arar, M. and C. Jung (2021). "Improving the Indoor Air Quality in Nursery Buildings in United Arab Emirates." Int J Environ Res Public Health **18**(22).
Araujo, O. R. and M. C. Araujo (2020). "The zero impact of the Vehicle Inspection Program on public health in São Paulo, SP." Rev Saude Publica **54**: 84.
Banerjee, R. and R. Maharaj (2020). "Heat, infant mortality, and adaptation: Evidence from India." Journal of Development Economics **143**: 102378.
Barkjohn, K. K., C. Norris, X. Cui, L. Fang, T. Zheng, J. J. Schauer, Z. Li, Y. Zhang, M. Black, J. J. Zhang and M. H. Bergin (2021). "Real-time measurements of PM(2.5) and ozone to assess the effectiveness of residential indoor air filtration in Shanghai homes." Indoor Air **31**(1): 74-87.
Barn, P., E. Gombojav, C. Ochir, B. Boldbaatar, B. Beejin, G. Naidan, J. Galsuren, B. Legtseg, T. Byambaa, J. A. Hutcheon, C. Janes, P. A. Janssen, B. P. Lanphear, L. C. McCandless, T. K. Takaro, S. A. Venners, G. M. Webster and R. W. Allen (2018). "The effect of portable HEPA filter air cleaner use during pregnancy on fetal growth: The UGAAR randomized controlled trial." Environment International **121**: 981-989.
Barn, P., E. Gombojav, C. Ochir, B. Laagan, B. Beejin, G. Naidan, B. Boldbaatar, J. Galsuren, T. Byambaa, C. Janes, P. A. Janssen, B. P. Lanphear, T. K. Takaro, S. A. Venners, G. M. Webster, W. Yuchi, C. D. Palmer, P. J. Parsons, Y. M. Roh and R. W. Allen (2018). "The effect of portable HEPA filter air cleaners on indoor PM(2.5) concentrations and second hand tobacco smoke exposure among pregnant women in Ulaanbaatar, Mongolia: The UGAAR randomized controlled trial." Sci Total Environ **615**: 1379-1389.
Brehmer, C., C. Norris, K. K. Barkjohn, M. H. Bergin, J. Zhang, X. Cui, Y. Zhang, M. Black, Z. Li, M. Shafer and J. J. Schauer (2019). "The impact of household air cleaners on the chemical composition and children's exposure to PM(2.5) metal sources in suburban Shanghai." Environ Pollut **253**: 190-198.
Castell, N., P. Schneider, S. Grossberndt, M. F. Fredriksen, G. Sousa-Santos, M. Vogt and A. Bartonova (2018). "Localized real-time information on outdoor air quality at kindergartens in Oslo, Norway using low-cost sensor nodes." Environmental Research **165**: 410-419.
Castro, A., N. Künzli and T. Götschi (2017). "Health benefits of a reduction of PM10 and NO2 exposure after implementing a clean air plan in the Agglomeration Lausanne-Morges." International Journal of Hygiene and Environmental Health **220**(5): 829-839.
De La Iglesia, D. H., J. F. De Paz, G. Villarrubia González, A. L. Barriuso and J. Bajo (2018). "A Context-Aware Indoor Air Quality System for Sudden Infant Death Syndrome Prevention." Sensors (Basel) **18**(3).
DeCicca, P. and N. Malak (2020). "When good fences aren't enough: The impact of neighboring air pollution on infant health." Journal of Environmental Economics and Management **102**: 102324.
Dirks, K. N., J. A. Salmond and N. Talbot (2018). "Air Pollution Exposure in Walking School Bus Routes: A New Zealand Case Study." Int J Environ Res Public Health **15**(12).
Enkhbat, U., E. Gombojav, C. Banzrai, S. Batsukh, B. Boldbaatar, E. Enkhtuya, D. C. Bellinger, B. P. Lanphear, L. C. McCandless and R. W. Allen (2022). "Portable HEPA filter air cleaner use during pregnancy and children's autistic behaviors at four years of age: The UGAAR randomized controlled trial." Environment International **168**: 107432.
Enkhbat, U., E. Gombojav, C. Banzrai, S. Batsukh, B. Boldbaatar, E. Enkhtuya, C. Ochir, D. C. Bellinger, B. P. Lanphear, L. C. McCandless and R. W. Allen (2021). "Portable HEPA filter air cleaner use during pregnancy and children's behavior problem scores: a secondary analysis of the UGAAR randomized controlled trial." Environmental Health **20**(1): 78.
Estrella, B., F. Sempértegui, O. H. Franco, M. Cepeda and E. N. Naumova (2019). "Air pollution control and the occurrence of acute respiratory illness in school children of Quito, Ecuador." J Public Health Policy **40**(1): 17-34.

Gao, X., Y. Xu, Y. Cai, J. Shi, F. Chen, Z. Lin, T. Chen, Y. Xia, W. Shi and Z. Zhao (2019). "Effects of filtered fresh air ventilation on classroom indoor air and biomarkers in saliva and nasal samples: A randomized crossover intervention study in preschool children." Environmental Research **179**: 108749.

Gehrsitz, M. (2017). "The effect of low emission zones on air pollution and infant health." Journal of Environmental Economics and Management **83**: 121-144.

Giannadaki, D., J. Lelieveld and A. Pozzer (2016). "Implementing the US air quality standard for PM2.5 worldwide can prevent millions of premature deaths per year." Environ Health **15**(1): 88.

Griffiths, C. J., I. Mudway, H. Wood, N. Marlin, I. Dundas, R. Walton, J. Grigg, A. Sheikh, T. Lee and F. Kelly (2016). "P180 Impact of the london low emission zone on children's respiratory health: a sequential yearly cross sectional study 2008–2014." Thorax **71**(Suppl 3): A182.

Hartinger, S. M., N. Nuño, J. Hattendorf, H. Verastegui, W. Karlen, M. Ortiz and D. Mäusezahl (2020). "A factorial cluster-randomised controlled trial combining home-environmental and early child development interventions to improve child health and development: rationale, trial design and baseline findings." BMC Med Res Methodol **20**(1): 73.

Heo, S., A. Nori-Sarma, K. Lee, T. Benmarhnia, F. Dominici and M. L. Bell (2019). "The Use of a Quasi-Experimental Study on the Mortality Effect of a Heat Wave Warning System in Korea." Int J Environ Res Public Health **16**(12).

Heydon, J. and R. Chakraborty (2020). "Can portable air quality monitors protect children from air pollution on the school run? An exploratory study." Environmental Monitoring and Assessment **192**(3): 195.

Inoue, T., N. Nunokawa, D. Kurisu and K. Ogasawara (2020). "Particulate air pollution, birth outcomes, and infant mortality: Evidence from Japan's automobile emission control law of 1992." SSM - Population Health **11**: 100590.

Jasemzadeh, M., M. A. Khafaie, N. Jaafarzadeh and M. Araban (2018). "Effectiveness of a theory-based mobile phone text message intervention for improving protective behaviors of pregnant women against air pollution: a randomized controlled trial." Environ Sci Pollut Res Int **25**(7): 6648-6655.

Jhun, I., J. M. Gaffin, B. A. Coull, M. F. Huffaker, C. R. Petty, W. J. Sheehan, S. N. Baxi, P. S. Lai, C. M. Kang, J. M. Wolfson, D. R. Gold, P. Koutrakis and W. Phipatanakul (2017). "School Environmental Intervention to Reduce Particulate Pollutant Exposures for Children with Asthma." J Allergy Clin Immunol Pract **5**(1): 154-159.e153.

Kaviany, P., E. P. Brigham, J. M. Collaco, J. L. Rice, H. Woo, M. Wood, R. Koehl, T. D. Wu, M. N. Eakin, K. Koehler, N. N. Hansel and M. C. McCormack (2022). "Patterns and predictors of air purifier adherence in children with asthma living in low-income, urban households." J Asthma **59**(5): 946-955.

Kim, H. K. and G. H. Jeong (2022). "Effect of Pro-Environmental Prenatal Education Program on Pregnant Women's Environmental Health Awareness and Behaviors based on the Protection Motivation Theory." Inquiry **59**: 469580211047045.

Kouis, P., E. Michailidi, S. I. Papatheodorou, M. G. Kakkoura, E. Dimitriou, P. Koutrakis, E. Galanakis and P. K. Yiallouros (2019). "Mitigating the health effects of Desert Dust Storms through exposure reduction approaches: The LIFE MEDEA Asthma study." European Respiratory Journal **54**(suppl 63): PA2831.

Lanza, K., M. Alcazar, D. M. Hoelscher and H. W. Kohl (2021). "Effects of trees, gardens, and nature trails on heat index and child health: design and methods of the Green Schoolyards Project." BMC Public Health **21**(1): 98.

Lee, E., A. Dressel, J. Bell-Calvin, Z. Garacci and J. Steinberg (2020). "Assessment of Indoor Air Quality and Cleaning Behaviors in Urban Child Care Facilities." Journal of Allergy and Clinical Immunology **145**(2): AB83.

Lee, G. H., J. H. Kim, S. Kim, S. Lee and D. H. Lim (2020). "Effects of Indoor Air Purifiers on Children with Asthma." Yonsei Med J **61**(4): 310-316.

Li, Y., B. Sun, C. Yang, X. Zhuang, L. Huang, Q. Wang, P. Bi, Y. Wang, X. Yao and Y. Cheng (2022). "Effectiveness Evaluation of a Primary School-Based Intervention against Heatwaves in China." Int J Environ Res Public Health **19**(5).

MacNeill, M., N. Dobbin, M. St-Jean, L. Wallace, L. Marro, T. Shin, H. You, R. Kulka, R. W. Allen and A. J. Wheeler (2016). "Can changing the timing of outdoor air intake reduce indoor concentrations of traffic-related pollutants in schools?" Indoor Air **26**(5): 687-701.

Maher, B. A., T. Gonet, V. V. Karloukovski, H. Wang and T. J. Bannan (2022). "Protecting playgrounds: local-scale reduction of airborne particulate matter concentrations through particulate deposition on roadside 'tredges' (green infrastructure)." Sci Rep **12**(1): 14236.
Maji, K. J., M. Arora and A. K. Dikshit (2018). "Premature mortality attributable to PM2.5 exposure and future policy roadmap for 'airpocalypse' affected Asian megacities." Process Safety and Environmental Protection **118**: 371-383.
Martenies, S. E. and S. A. Batterman (2018). "Effectiveness of Using Enhanced Filters in Schools and Homes to Reduce Indoor Exposures to PM(2.5) from Outdoor Sources and Subsequent Health Benefits for Children with Asthma." Environ Sci Technol **52**(18): 10767-10776.
Moreno-Rangel, A., J. Baek, T. Roh, X. Xu and G. Carrillo (2020). "Assessing Impact of Household Intervention on Indoor Air Quality and Health of Children with Asthma in the US-Mexico Border: A Pilot Study." Journal of Environmental and Public Health **2020**: 6042146.
Mudway, I. S., I. Dundas, H. E. Wood, N. Marlin, J. B. Jamaludin, S. A. Bremner, L. Cross, A. Grieve, A. Nanzer, B. M. Barratt, S. Beevers, D. Dajnak, G. W. Fuller, A. Font, G. Colligan, A. Sheikh, R. Walton, J. Grigg, F. J. Kelly, T. H. Lee and C. J. Griffiths (2019). "Impact of London's low emission zone on air quality and children's respiratory health: a sequential annual cross-sectional study." The Lancet Public Health **4**(1): e28-e40.
Niu, J., Q. Qu, J. Li, X. Liu, B. Zhang, Z. Li, G. Ding, Y. Sun, Y. Shi, Y. Wan, X. Hu, L. C. Chen, A. Mendelsohn, Y. Chen and L. Trasande (2015). "Improving Knowledge about Children's Environmental Health in Northwest China." Int J Environ Res Public Health **13**(1): 80.
Park, H. K., K. C. Cheng, A. O. Tetteh, L. M. Hildemann and K. C. Nadeau (2017). "Effectiveness of air purifier on health outcomes and indoor particles in homes of children with allergic diseases in Fresno, California: A pilot study." J Asthma **54**(4): 341-346.
Parker, R., A. Whitehouse, T. Hadaway, R. Araujo, C. McCowlen, E. Foord and J. Grigg (2021). Let's talk air pollution – it's everyones responsibility.
Patel, D., T. Shibata, J. Wilson and A. Maidin (2016). "Challenges in evaluating PM concentration levels, commuting exposure, and mask efficacy in reducing PM exposure in growing, urban communities in a developing country." Sci Total Environ **543**(Pt A): 416-424.
Payne-Sturges, D. C., M. A. Marty, F. Perera, M. D. Miller, M. Swanson, K. Ellickson, D. A. Cory-Slechta, B. Ritz, J. Balmes, L. Anderko, E. O. Talbott, R. Gould and I. Hertz-Picciotto (2019). "Healthy Air, Healthy Brains: Advancing Air Pollution Policy to Protect Children's Health." Am J Public Health **109**(4): 550-554.
Perera, F., D. Cooley, A. Berberian, D. Mills and P. Kinney (2020). "Co-Benefits to Children's Health of the U.S. Regional Greenhouse Gas Initiative." Environmental Health Perspectives **128**(7): 077006.
Pormosayebi, S., M. Shamsi, M. Khorsandi, A. Koolivand and M. Ranjbaran (2018). "The effect of training based on health belief model (HBM) in preventing exposure to polluted air in pregnant women." Journal of Babol University of Medical Sciences **20**: 57-63.
Riederer, A. M., J. E. Krenz, M. I. Tchong-French, E. Torres, A. Perez, L. R. Younglove, K. L. Jansen, D. C. Hardie, S. A. Farquhar, P. D. Sampson and C. J. Karr (2021). "Effectiveness of portable HEPA air cleaners on reducing indoor PM(2.5) and NH(3) in an agricultural cohort of children with asthma: A randomized intervention trial." Indoor Air **31**(2): 454-466.
Riederer, A. M., J. E. Krenz, M. I. Tchong-French, E. Torres, A. Perez, L. R. Younglove, K. L. Jansen, D. C. Hardie, S. A. Farquhar, P. D. Sampson, N. Metwali, P. S. Thorne and C. J. Karr (2021). "Effectiveness of portable HEPA air cleaners on reducing indoor endotoxin, PM(10,) and coarse particulate matter in an agricultural cohort of children with asthma: A randomized intervention trial." Indoor Air **31**(6): 1926-1939.
Rufo, J. C., J. Madureira, I. Paciência, L. Aguiar, J. P. Teixeira, A. Moreira and E. de Oliveira Fernandes (2016). "Indoor air quality and atopic sensitization in primary schools: A follow-up study." Porto Biomed J **1**(4): 142-146.
Rumchev, K., A. Lee, B. Maycock and J. Jancey (2021). "Reducing car idling at primary schools: An intervention study of parent behaviour change in Perth, Western Australia." Health Promotion Journal of Australia **32**(3): 383-390.
Sá, J. P., P. Branco, M. C. M. Alvim-Ferraz, F. G. Martins and S. I. V. Sousa (2017). "Evaluation of Low-Cost Mitigation Measures Implemented to Improve Air Quality in Nursery and Primary Schools." Int J Environ Res Public Health **14**(6).

Shortridge, A., W. Walker Vi, D. D. White, M. M. Guardaro, D. M. Hondula and J. K. Vanos (2022). "HeatReady schools: A novel approach to enhance adaptive capacity to heat through school community experiences, risks, and perceptions." Climate Risk Management **36**: 100437.
Smart, N. R., C. J. Horwell, T. S. Smart and K. S. Galea (2020). "Assessment of the Wearability of Facemasks against Air Pollution in Primary School-Aged Children in London." Int J Environ Res Public Health **17**(11).
Spencer-Hwang, R., S. Soret, J. Valladares, X. Torres, M. Pasco-Rubio, M. Dougherty, W. Kim and S. Montgomery (2016). "Strategic Partnerships for Change in an Environmental Justice Community: The ENRRICH Study." Prog Community Health Partnersh **10**(4): 541-550.
Tamana, S. K., E. Gombojav, A. Kanlic, C. Banzrai, S. Batsukh, E. Enkhtuya, B. Boldbaatar, B. P. Lanphear, S. A. Lear, L. C. McCandless, S. A. Venners and R. W. Allen (2021). "Portable HEPA filter air cleaner use during pregnancy and children's body mass index at two years of age: The UGAAR randomized controlled trial." Environ Int **156**: 106728.
Ulziikhuu, B., E. Gombojav, C. Banzrai, S. Batsukh, E. Enkhtuya, B. Boldbaatar, D. C. Bellinger, B. P. Lanphear, L. C. McCandless, S. K. Tamana and R. W. Allen (2022). "Portable HEPA Filter Air Cleaner Use during Pregnancy and Children's Cognitive Performance at Four Years of Age: The UGAAR Randomized Controlled Trial." Environ Health Perspect **130**(6): 67006.
Vervoort, R., B. Blocken and T. van Hooff (2019). "Reduction of particulate matter concentrations by local removal in a building courtyard: Case study for the Delhi American Embassy School." Science of The Total Environment **686**: 657-680.
Willis, M. D., E. L. Hill, M. L. Kile, S. Carozza and P. Hystad (2020). "Assessing the effectiveness of vehicle emission regulations on improving perinatal health: a population-based accountability study." International Journal of Epidemiology **49**(6): 1781-1791.
Yang, M. and S.-Y. Chou (2018). "The impact of environmental regulation on fetal health: Evidence from the shutdown of a coal-fired power plant located upwind of New Jersey." Journal of Environmental Economics and Management **90**: 269-293.
Zaeh, S. E., K. Koehler, M. N. Eakin, C. Wohn, I. Diibor, T. Eckmann, T. D. Wu, D. Clemons-Erby, C. E. Gummerson, T. Green, M. Wood, E. Majd, M. L. Stein, A. Rule, M. F. Davis and M. C. McCormack (2021). "Indoor Air Quality Prior to and Following School Building Renovation in a Mid-Atlantic School District." Int J Environ Res Public Health **18**(22).
Zauli-Sajani, S., S. Marchesi, G. Boselli, E. Broglia, A. Angella, E. Maestri, N. Marmiroli and A. Colacci (2022). "Effectiveness of a Protocol to Reduce Children's Exposure to Particulate Matter and NO(2) in Schools during Alert Days." Int J Environ Res Public Health **19**(17)

Supplementary File 3: Summary table of included grey literature (n=8) reporting on interventions on climate hazards (n=14)

Author, year [country]	Climate Hazard [specifics]	Aim/objective(s)	Population of interest	Focus of intervention [specifics]	Intervention description	Method of evaluation / data analysis	Summary of findings on MNCH / intervention outcomes measured
City Resilience Toolkit - Response to Deadly Heat Waves and Preparing for Rising Temperatures<br><br>(Natural Resources Defense Council 2016)<br><br>India	Extreme heat	To protect the city population from extreme heat	Children<br>Parents / carers	Provision of educational information (including preventative behaviours) to parents, families, carers, teachers<br><br>Awareness raising and education<br><br>Modifications to building structure, layout, and building materials to enhance thermal comfort and mitigate extreme heat indoors	A How-to-Manual developed from multiple strands of an overall heat action plan in Ahmedabad that began in 2010 and included multiple interventions. These covered: city engagement through key stakeholder engagement, establishing vulnerabilities and heat-health threshold temperatures, developing a heat action plan, strengthening coordination efforts through capacity building, public awareness campaigns around what to do in extreme heat events, and structural changes such as 'cool roofs'.	No evaluation reported	Cost-effectiveness, impact on most vulnerable populations, health records during heat seasons, feedback from residents, levels or awareness and behaviour change, heat related morbidity and mortality
A Practical Guide to Cool Roofs and Cool Pavements	Extreme heat	To mitigate the effects of extreme heat through implementation of cool roofs	Children	Modifications to building structure, layout, and building	Installation of various cool roof materials (including elastomeric coatings, lime coatings and tiles) on different buildings around	No evaluation reported	Planned intervention with no results published, focused on energy saving and thermal comfort benefits of cool roofs

Author, year [country]	Climate Hazard [specifics]	Aim/objective(s)	Population of interest	Focus of intervention [specifics]	Intervention description	Method of evaluation / data analysis	Summary of findings on MNCH / intervention outcomes measured
(Global Cool Cities Alliance and R20 Regions of Climate Action 2012)<br><br>India				materials to enhance thermal comfort and mitigate extreme heat indoors	Delhi, including schools and hospitals		
Heatwave Guide for Cities<br><br>(International Federation of Red Cross and Red Crescent Societies 2020)<br><br>Viet Nam	Extreme Heat	To create forecast based actions for heatwaves	Children Parents / caregivers	Heat (early) warning system of extreme heat events<br><br>Knowledge, attitude, and practice related surveys<br><br>Modifications to building structure, layout, and building materials to enhance thermal comfort and mitigate extreme heat indoors	The intervention was based in Hanoi and involved co-creating heatwave forecasts, conducting a knowledge, attitude, and practice survey to understand the population's heat risk coping capacity, identifying potential partners and vulnerable population locations, and implementing early heat-health impact reduction actions such as establishing cooling centres, household retrofitting, and providing cooling fans with ice tanks for night-time use.	No evaluation reported	To develop the intervention, they used heatwave forecasts, capacity survey, stakeholder analysis, GIS mapping<br><br>The outcomes measured included understanding the population's capacity to cope with heat risks, locations of vulnerable people, and the effectiveness of the implemented early actions to reduce heat-health impacts
Heatwave Guide for Cities	Extreme Heat	Outline of an intervention implementing cooling sprays in parks	Children	Urban landscape management	The City of Cape Town installed six spray parks in recreational spaces in lower income areas of the city to provide cooling services for	No evaluation reported	The study involved identifying the heat risks in Cape Town, particularly in lower income areas, and implementing the installation of six spray parks as a cooling measure.

Author, year [country]	Climate Hazard [specifics]	Aim/objective(s)	Population of interest	Focus of intervention [specifics]	Intervention description	Method of evaluation / data analysis	Summary of findings on MNCH / intervention outcomes measured
(International Federation of Red Cross and Red Crescent Societies 2020)<br><br>South Africa					children of all ages, including those with disabilities, during heatwaves. These spray parks consume only 15-20% of the water used by a medium-sized municipal pool, making them a viable solution for water-stressed cities.		
Primer for Cool Cities: Reducing Excessive Urban Heat<br><br>(World Bank 2020)<br><br>France	Extreme Heat	Implementation of interventions to mitigate the effects of extreme heat in the way of the 2003 heat wave	Children<br>Parents / carers	Urban landscape management<br><br>Nature based solutions: (Re)-greening outdoor areas, especially those frequented by children, including schools and playgrounds<br><br>Digital intervention<br><br>Heat (early) warning system of extreme heat events	Paris has undertaken four initiatives to create areas of heat resiliency. First, the Cool Islands initiative aims to have every Parisian within a seven-minute walk of a 'cool island', which could be a swimming area, air-conditioned building, park, or water/misting feature. The city plans to have an additional 300 cool islands in place by 2030.<br><br>The second initiative, Urban Oasis, focuses on cooling schoolyards using shading, greenery, permeable surfaces, and cool pavements. The city received a US$5 million award to convert 30 cool schoolyards by 2019, with plans to complete all 770 Parisian schoolyards by 2040.	No evaluation reported	Locations of 'Cool Islands' in Paris, schools covered by 'Urban Oasis' scheme

Author, year [country]	Climate Hazard [specifics]	Aim/objective(s)	Population of interest	Focus of intervention [specifics]	Intervention description	Method of evaluation / data analysis	Summary of findings on MNCH / intervention outcomes measured
					Third, Cool Pathways links the network of cool islands to allow Parisians to minimize thermal discomfort on hot days. The plan involves enhancing pedestrian walkways with tree canopies or structures, and green, permeable and solar reflective pavements.<br><br>Lastly, Paris partnered with the National Observatory of Athens to launch a mobile application, Extrema, to help Parisians stay cool during extreme heat events.		
Heat and Health in WHO European Region - Updated Evidence for Effective Prevention<br><br>(World Health Organization 2021)<br><br>Greece, Spain, Italy, France, Netherlands	Extreme Heat	To inform individuals of their heat–health risk (no/low/increased/high risk), taking into account the user's profile characteristics (age, presence of chronic disease associated with a greater risk during heat-waves, use of medication), in real time.	Children	Digital intervention<br><br>Heat (early) warning system of extreme heat events	EXTREMA is a mobile application that informs individuals about their heat-health risk (no/ low/increased/high risk) in real time, taking into account user's profile characteristics such as age, presence of chronic disease, and use of medication. The app uses satellite thermal images and numerical weather predictions to estimate heat-health risks. It also provides health recommendations, information on cooling	No evaluation reported	Outcomes that the intervention sought to generate evidence on: Measures information on cool centres (opening hours, entrance fee, capacity), current and previous day alerts on weather information, real-time maps of extreme temperature

Author, year [country]	Climate Hazard [specifics]	Aim/objective(s)	Population of interest	Focus of intervention [specifics]	Intervention description	Method of evaluation / data analysis	Summary of findings on MNCH / intervention outcomes measured
					centres, and supports multiple profiles, allowing users to check on family members at multiple locations. Additionally, there's a web service dashboard for local municipalities to manage information on cooling centres.		
Heat and Health in WHO European Region - Updated Evidence for Effective Prevention<br><br>(World Health Organization 2021)<br><br>France	Extreme Heat	A national heat-wave plan to protect the population	Children<br>Parents / caregivers	Provision of educational information (including preventative behaviours) to parents, families, carers, teachers<br><br>Heat (early) warning system of extreme heat events	This was a complex intervention across various governance levels (national, regional), including: provision of training and educational materials for stakeholders and the population; distribution of extreme heat advice in summer; training and educational materials for health care professionals; the coordination at the national level of a heat warning system, evaluation of the heat-health action plan (HHAP); recommendations to reinforce hospitals in summer	No evaluation reported	Outcomes that the intervention sought to generate evidence on: Understanding of the health effects of heat, risk perception, knowledge of vulnerable groups, self-perception of risk, knowledge of prevention attitudes, follow-up of recommendations.
Beating the Heat - A Sustainable Cooling Handbook for Cities	Extreme Heat	Creation of a Heat Action Plan and warning system in Ahmedabad following a heatwave in 2010.	Children<br>Parents / caregivers	Heat (early) warning system of extreme heat events	The Heat Action Plan in Ahmedabad focuses on early warning systems, public awareness and community outreach, capacity-building among	No evaluation reported	Outcomes that the intervention sought to generative evidence on: temperature forecast, public awareness, heat exposure, health-care professionals knowledge and practice around heat-related illnesses.

Author, year [country]	Climate Hazard [specifics]	Aim/objective(s)	Population of interest	Focus of intervention [specifics]	Intervention description	Method of evaluation / data analysis	Summary of findings on MNCH / intervention outcomes measured
(United Nations Environment Programme 2021)<br><br>India				Awareness raising and education<br><br>Modifications to building structure, layout, and building materials to enhance thermal comfort and mitigate extreme heat indoors	medical professionals, and measures to reduce heat exposure. Specific interventions include sending text alerts during heat warnings, training healthcare professionals to recognize heat-related illnesses, and promoting passive cooling roofs. Public awareness and community outreach includes working with school children		
Beating the Heat - A Sustainable Cooling Handbook for Cities<br><br>(United Nations Environment Programme 2021)<br><br>India	Extreme Heat	Reduction in necessary electricity generation capacity and carbon emissions	Children<br>Parents / caregivers	Modifications to building structure, layout, and building materials to enhance thermal comfort and mitigate extreme heat indoors	The development of a high-density city district in Gujarat will incorporate a cooling system. The system includes stratified thermal energy storage tanks and will use an environmentally friendly refrigerant.	No evaluation reported	Reduction in necessary electricity generation capacity and carbon emissions
Beating the Heat - A Sustainable Cooling	Extreme Heat	To identify mitigation and adaptation strategies for extreme heat in	Children<br>Parents / caregivers	Awareness raising and education	Workshops to educate people on urban heat island effects, a mapping of community hot spots,	No evaluation reported	Heat action plans included: reimagining bus stops, prioritizing water features, street trees and shading.

Author, year [country]	Climate Hazard [specifics]	Aim/objective(s)	Population of interest	Focus of intervention [specifics]	Intervention description	Method of evaluation / data analysis	Summary of findings on MNCH / intervention outcomes measured
Handbook for Cities<br><br>(United Nations Environment Programme 2021)<br><br>USA		Maricopa County, USA			creation of heat action plans		
Healthy Environments for Healthy Children - Global Programme Framework<br><br>(UNICEF 2021)<br><br>Mongolia	Air pollution	Research, advocacy, and training to mitigate the impact of air pollution	Children	Awareness raising and education<br><br>Policy advocacy	Multiple strategies were developed to mitigate the effects of air pollution in winter, including research and evidence generation on risks, policy advocacy and public awareness work, integrated community-based management of air pollution effects on children's health in training for health practitioners	No evaluation reported	The intervention sought to promote community-based management of childhood illnesses and mitigate air pollution impacts on child health
Reducing Urban Heat Islands to Protect Health in Canada<br><br>Health Canada (Health Canada 2020)<br><br>Canada	Extreme Heat	An intervention in Québec to mitigate extreme heat through reductions in urban heat islands	Children	Nature based solutions: (Re)-greening outdoor areas, especially those frequented by children, including schools and playgrounds	The pilot intended to 'green' Anna Street, which was selected due to its lack of greenery and its location near a school. Greening included planting trees, creating rain gardens, and installing permeable surfaces	No evaluation reported	Outcomes measured by trees planted, reduction of asphalt surfaces reserved for cars, number of new planting bays, tree canopy increase

Author, year [country]	Climate Hazard [specifics]	Aim/objective(s)	Population of interest	Focus of intervention [specifics]	Intervention description	Method of evaluation / data analysis	Summary of findings on MNCH / intervention outcomes measured
				Urban landscape management			
Reducing Urban Heat Islands to Protect Health in Canada<br><br>Health Canada (Health Canada 2020)<br><br>Canada	Extreme Heat	An intervention in Toronto to mitigate extreme heat through reductions in urban heat islands	Children	Nature based solutions: (Re)-greening outdoor areas, especially those frequented by children, including schools and playgrounds<br><br>Urban landscape management	The intervention aimed to increase shade across the city, primarily focused in areas frequented by children. This included through natural tree canopy or constructed coverings	No evaluation reported	Measured in reductions in exposure to ultraviolet radiation and associated health risks through shade
Reducing Urban Heat Islands to Protect Health in Canada<br><br>Health Canada (Health Canada 2020)<br><br>Canada	Extreme Heat	An intervention in Ontario in Canadian cities to mitigate extreme heat through reductions in urban heat islands	Children	Nature based solutions: (Re)-greening outdoor areas, especially those frequented by children, including schools and playgrounds<br><br>Urban landscape management	Aimed to improve thermal comfort in parks and playgrounds, including installing shade structures, water fountains, and planting trees.	No evaluation reported	Measured in share structures built, shaded seating areas developed, splash pads installed, water-bottle refilling sites, and tree planting

Global Cool Cities Alliance and R20 Regions of Climate Action (2012). A Practical Guide to Cool Roofs and Cool Pavements, Global Cool Cities Alliance, R20 Regions of Climate Action.
Health Canada (2020). Reducing Urban Heat Islands To Protect Health in Canada. Ottawa, Canada, Health Canada.
International Federation of Red Cross and Red Crescent Societies (2020). Heatwave Guide for Cities, IFRC.
Natural Resources Defense Council (2016). City resilience toolkit: response to deadly heat waves and preparing for rising temperatures
.
UNICEF (2021). Healthy Environments for Healthy Children: Global Programme Framework. New York, USA, UNICEF.
United Nations Environment Programme (2021). Beating the Heat: A Sustainable Cooling Handbook for Cities. Nairobi, Kenya, UNEP.
World Bank (2020). Primer for Cool Cities: Reducing Excessive Urban Heat–With a Focus on Passive Measures. Washington DC, USA, World Bank.
World Health Organization (2021). "Heat and health in the WHO European Region: updated evidence for effective prevention."
